# Adherence to ARRIVE Guidelines in Chinese Journal Reports on Neoplasms in Animals

**DOI:** 10.1371/journal.pone.0154657

**Published:** 2016-05-16

**Authors:** Yali Liu, Xingxing Zhao, Yuefen Mai, Xinxin Li, Jin Wang, Lili Chen, Jing Mu, Gengxue Jin, Hongping Gou, Wanting Sun, Yuchen Feng

**Affiliations:** 1 Evidence Based Medicine Center, School of Basic Medical Sciences, Lanzhou University, Lanzhou, China; 2 Key Laboratory of Evidence Based Medicine and Knowledge Translation of Gansu Province, Lanzhou, China; 3 Institute of Basic Research in Clinical Medicine, China Academy of Chinese Medical Sciences, Dongzhimen, Beijing, China; 4 Peking University Health Science Center, Beijing, China; 5 The Second Clinical Medical College of Lanzhou University, Lanzhou, China; 6 The First Clinical Medical College of Lanzhou University, Lanzhou, China; Cardiff University, UNITED KINGDOM

## Abstract

**Background:**

The Animals in Research: Reporting *In Vivo* Experiments (ARRIVE) guidelines were published in 2010 with the aim of improving the quality of studies involving animals. However, how well Chinese studies involving animal neoplasms adhere to these guidelines has not been assessed.

**Objective:**

To evaluate the reporting quality of such experiments published between 2010 and 2012 in Chinese journals with support from the National Natural Science Foundation of China.

**Methods:**

We searched the Chinese Science Citation and Chinese Journal Full-Text Databases for articles published between 2010 and 2012 involving neoplasms in animals. The data were extracted into pre-prepared forms. Reporting quality was assessed using the ARRIVE checklist—39 items plus information on blinding.

**Results:**

Three hundred and ninety-six animal studies were included in the analysis: 127 studies published in 2010, 140 studies published in 2011, and 129 studies published in 2012. The range of ARRIVE score is from 12 to 27 with a maximum possible score of 40. Studies published in 2012 (*P* = 0.012), 2011 (*P* = 0.015), 2010, July~Dec (*P*<0.017) had a significantly larger ARRIVE checklist score than those published in Jan.~June, 2010, respectively.

**Conclusions:**

Experiments involving neoplasms in animals published in Chinese journals generally have not comprehensively reported the information recommended by the ARRIVE guidelines. We strongly recommend that researchers conducting such studies report this information.

## Introduction

Animals are often used during biomedical research studies, but such studies are often controversial because there are many differences between animals and humans. The aims of preclinical animal experiments are to perform preliminary safety and efficacy validations of the (new) intervention under study, with the results determining whether the intervention should be further assessed in clinical studies and trials. Every year, many animal experiments, including those supported by foundations and other organizations, are published in a variety of journals. In China, the National Natural Science Foundation of China (NSFC) is one of the major funding sources for basic science research.

Although random allocation and blinding are common in clinical trials, they are not common in animal experiments that precede clinical trials. One study[1] assessed 290 published animal experiments and reported that 32.41% (94/290) used randomization and 9.66% (28/290) used blinding. Non-randomized, non-blinded animal studies are more likely to report a difference between study groups than animal studies that use these methods[1]. Experimental design, statistical analysis, and reporting issues have also been found for animal studies [2,3].

The Animals in Research: Reporting *In Vivo* Experiments (ARRIVE) guidelines published in 2010 were developed to improve the transparency and accuracy of bioscience research reporting. The guidelines detail the minimum information that should be reported when using animals in a research study and include a 20-item checklist[4]. In 2011, the ARRIVE guidelines were introduced in China[5]. To date, no study has assessed whether animal experiments of neoplasms published in Chinese journals adhere to these guidelines.

The aim of this study was to determine the reporting quality of experiments, specifically those involving neoplasms in animals, which were published in Chinese journals between 2010 and 2012 and were supported by the NSFC.

## Methods

### Inclusion/exclusion criteria

The targeted studies were those involving neoplasms in living rodents, including rats, mice, nude mice, and guinea pigs (Mammalia: Rodentia), published between 2010 and 2012 in Chinese journals, and supported by the NSFC.

We excluded studies on animals that were dead before the start of the experiment, when the studies were *invitro*, when no intervention was performed and no control group was included, when the studies focused on precancerosis, or when the studies were part of an academic dissertation or review article.

### Search strategy

We comprehensively and systematically searched the Chinese Science Citation Databases (CSCD) and the Chinese Journal Full-Text Databases (CJFD) on July 13, 2012 for all entries submitted from January 2010 to June 2012, and on June 19, 2013 for all entries submitted from July 2012 to December 2012. The main search terms were “neoplasm,” “animal experiment, *in vivo* experiment, basic research,” and “National Natural Science Foundation.” The search strategy is presented in S1 File.

### Screening

The titles and abstracts were independently screened by at least two reviewers (Xingxing Zhao, Yuefeng Mai, Xinxin Li, and/or Wanting Sun). Then, the full texts of potentially suitable articles were retrieved based on the inclusion and exclusion criteria. Disagreements concerning the suitability of an article were resolved by group discussions.

### Data extraction

Extraction into a pilot-tested standardized data form based on the ARRIVE guidelines was performed independently by at least two reviewers (Xingxing Zhao, Yuefeng Mai, Xinxin Li, Jin Wang, Lili Chen, Jing Mu, Gengxue Jin, and/or Hongping Gou). Inconsistencies were subsequently resolved by discussion among two or more reviewers, or the principal investigator Yali Liu made the final decision. The form consisted of two sections: (1) general characteristics (publication time, role of first author, the condition of interest, the allocation method of the included animals, and the number of funding organizations), (2) the ARRIVE information (39 items), and information about blinding that was separated from the checklist as item 6f and written as “Describe the information: If done, describe who was blinded (for example, outcome assessors) and how”, i.e., for a total of 40 scored items. Each item was assessed as “yes” (the item was described in the study) or “no” (the item was not described in the study).

### Data analysis

The data were summarized using Microsoft Excel (Version 2007; http://office.microsoft.com/zh-cn) and SPSS software (Version 21.0; http://www.spss.com). For continuous variables (for example the ARRIVE checklist scores), we expressed results as median and quartile (P_25_, P_45_) and comprised them for Kruskal-Wallis H Test. We use the traditional 0.05 definition of significance. The Bonferroni method was used for correcting for multiple comparisons. We mainly focus on a contrastive analysis of animal experiments before and after the publication of ARRIVE guidelines. We made 3 comparisons, then the new threshold is 0.017 (0.05/3).For categorical variables (for example the reported rate of ARRIVE checklist items), we expressed result as frequently and comprised them for the chi-square test.

## Results

### The literature search

**Fig 1:**
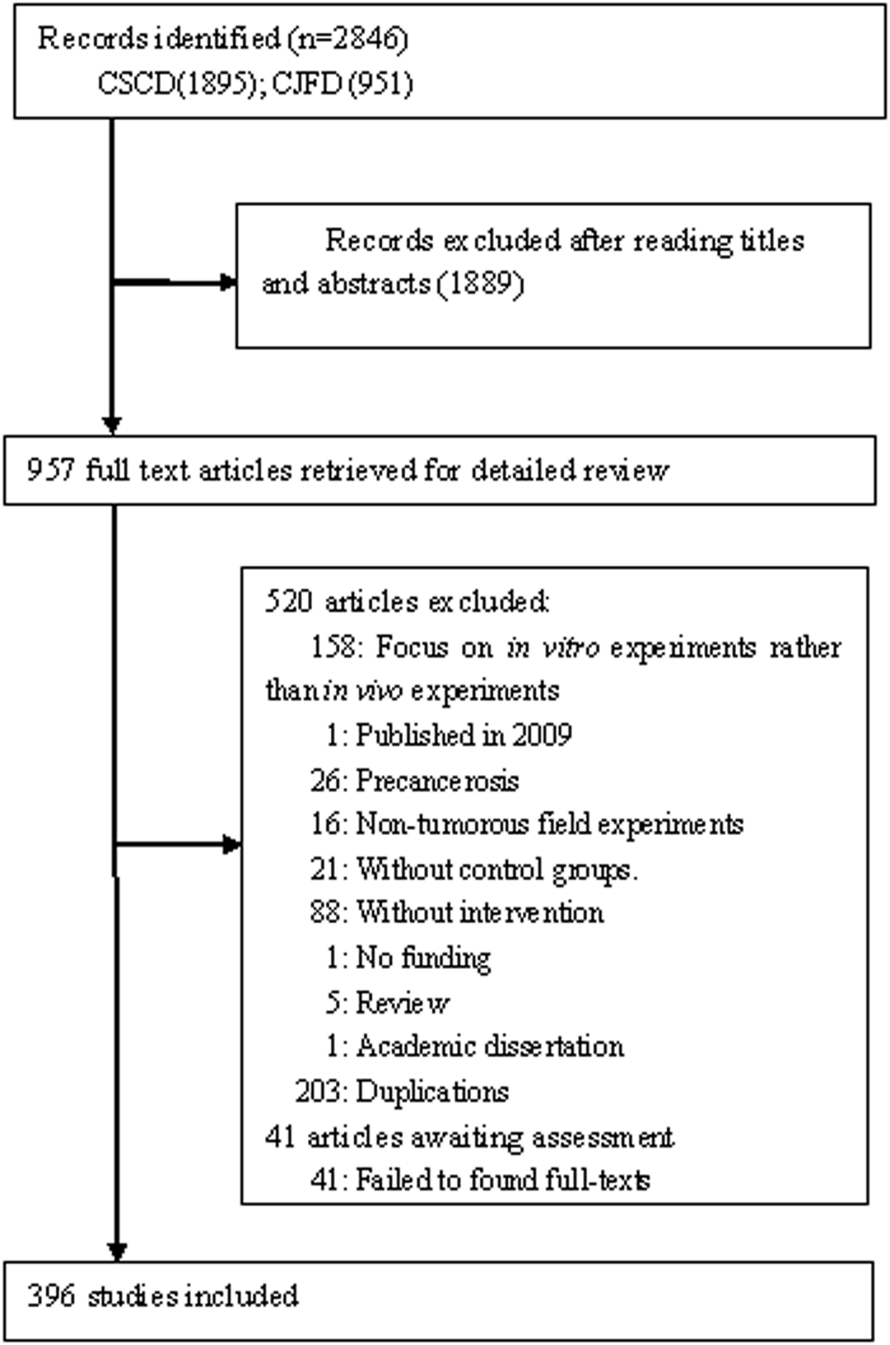
Flow chart of articles identified, included and exclude.

We initially identified 2846 studies. Of these, 1889 were excluded given their title or abstract, and a further 520 were excluded after an assessment of their full texts because they did not meet the inclusion criteria. The full texts of an additional 41 articles were not available. The remaining 396 studies met the inclusion criteria and were included in the analysis (Fig 1 and S2 File).

### General Characteristics of the Included Studies

The characteristics of the included studies are shown in Table 1. Of these studies, 126 (31.82%), 141 (35.61%), and 129 (32.58%) were published in 2010, 2011, and 2012, respectively. There were 77 studies which were published the release of the before ARRIVE guideline. For slightly more than half of the studies (219/396; 55.30%), the first author was affiliated with a hospital. The most commonly studied conditions were liver and lung cancers. The majority of the studies randomly assigned animals to the experimental or control group, but only 26.77% (106/396) described the randomization method in detail. Eleven studies used a random number table to perform randomization. Other randomization methods are described in Table 2.A total of 159 studies (40.15%) were supported by only the NSFC. The other 237 studies (59.85%) were supported by up to five sources.

**Table 1:** Characteristics of included studies.

Category	Characteristic	Number of n = 396 (%)
Publication year	2010	126(31.82)
	2011	141(35.61)
	2012	129(32.58)
Institution of first author	Hospitals	219(55.308)
	Medical University or College	137(34.60)
	Research institutions	50(12.63)
Condition focused on in the studies	Liver Cancer	79(19.95)
	Lung cancer	52(13.13)
	Bone cancer	32(8.08)
	Breast cancer	31(7.83)
	Stomach cancer	30(7.58)
	Intestinal cancer	29(7.32)
	Cervical cancer	8(2.02)
	Leukemia	7(1.77)
	Others	128(32.32)
The allocation method of included animals	Random allocation	355(89.65)
	Random number table	23 (6.48^@^)
	Computer generated random	1 (0.28^@^)
	Completely randomized design	1 (0.28^@^)
	Randomized block design	1 (0.28^@^)
	Sortition	1 (0.28^@^)
	Non-random allocation	41(10.35)
The number of funding	1	100 (100)
	2	159 (40.15)
	3	132 (33.33)
	4	75 (18.94)
	>4	30 (7.58)
Provided declarations of interest	Reported no conflicts of interests	2 (0.51)

^@ n = 355^

**Table 2:** Reporting of checklists for ARRIVE Guidelines.

Item	Sub-item	Number		Total Number (%) of n = 396	2010y. Jan.~June (%) n = 72	2010y July~Dec. (%) n = 55	2011y Number (%) n = 140	2012y Number (%) n = 129
TITLE		1	Provide as accurate and concise a description of the content of the article as possible	396(100)	72(100)	55(100)	140(100)	129(100)
ABSTRACT		2	Provide an accurate summary of the background, research objectives, including details of the species or strain of animal used, key methods, principal findings and conclusions of the study.	394(99.49)	71(98.61)	55(100)	140(100)	128(99.22)
INTRODUCTION								
	Background	3a	a. Include sufficient scientific background (including relevant references to previous work) to understand the motivation and context for the study, and explain the experimental approach and rationale.	388(97.98)	71(98.61)	52(94.55)	137(97.86)	128(99.22)
		3b	b. Explain how and why the animal species and model being used can address the scientific objectives and, where appropriate, the study’s relevance to human biology.	58(14.65)	8(11.11)	10(18.18)	22(15.71)	18(13.95)
	Objectives	4	Clearly describe the primary and any secondary objectives of the study, or specific hypotheses being tested	396(100)	72(100)	55(100)	140(100)	129(100)
METHODS								
	Ethical statement	5	Indicate the nature of the ethical review permissions, relevant licences (e.g. Animal [Scientific Procedures] Act 1986),and national or institutional guidelines for the care and use of animals, that cover the research.	7(1.77)	1(1.39)	1(1.82)	4(2.86)	1(0.78)
	Study design		*For each experiment*, *give brief details of the study design including*:					
		6a	a. The number of experimental and control groups.	392(98.99)	71(98.61)	55(100)	139(99.29)	127(98.45)
		6b	b. Any steps taken to minimise the effects of subjective bias when allocating animals to treatment (e.g.randomisation procedure).	363(91.67)	66(91.67)	48(87.27)	130(92.86)	119(92.25)
		6c	c. The experimental unit (e.g. a single animal, group, or cage of animals).	394(99.50)	72(100)	55(100)	140(100)	127(98.45)
		6d	d.A time-line diagram or flow chart can be useful to illustrate how complex study designs were carried out.	0(0)	0(0)	0(0)	0(0)	0(0)
		6f	f. If done, describe who was blinded (for example, outcome assessors) and how	1(0.25)	0(0)	0(0)	0 (0)	1(0.78)
	Experimental procedures	7a	How (e.g. drug formulation and dose, site and route of administration, anaesthesia and analgesia used [including monitoring], surgical (9procedure,method of euthanasia). Provide details of any specialist equipment used, including supplier(s).	396(100)	72(100)	55(100)	140(100)	129(100)
		7b	When (e.g. time. e of day).	342(86.36)	58(80.56)	53(96.36)	117(83.57)	114(88.37)
		7c	c.Where (e.g. home cage, laboratory, water maze	92(23.23)	9(12.50)	10(18.18)	26(18.57)	47(36.43)
		7d	d.Why (e.g. rationale for choice of specific anaesthetic, route of dministration, drug dose used)	0(0)	0(0)	0(0)	0(0)	0(0)
	Experimental animals	8a	a.Provide details of the animals used, including species, strain, sex, developmental stage (e.g. mean or median age plus age range) and weight (e.g. mean or median weight plus weight range)	383(96.72)	70(97.22)	54(98.18)	138(98.57)	121(93.80)
		8b	b.Provide further relevant information such as the source of animals, international strain nomenclature, genetic modification status (e.g. knock-out or transgenic), genotype, health/immune status, drug or test naive, previous procedures, etc.	295(74.49)	51(70.83)	42(76.36)	102(72.86)	100(77.52)
	Housing and husbandry	9a	a.Housing (e.g. type of facility,e.g. specific pathogen free [SPF]; type of cage or housing; bedding material; number of cage companions; tank shape and material etc. for fish).	198(50)	28(38.89)	24(43.64)	70(50)	76(58.91)
		9b	b. Husbandry conditions (e.g. breeding programme, light/dark cycle, temperature, quality of water etc. for fish, type of food, access to food and water, environmental enrichment).	96(24.24)	13(18.06)	13(23.64)	34(24.29)	36(27.91)
		9c	c.Welfare-related assessments and interventions that were carried out prior to, during, or after the experiment.	8(2.02)	2(2.78)	0(0)	0	6(4.65)
	Sample size	10a	a.Specify the total number of animals used in each experiment and the number of animals in each experimental group.	277(69.95)	44(61.11)	41(74.55)	92(65.71)	100(77.52)
		10b	b. Explain how the number of animals was arrived at. Provide details of any sample size calculation used.	0(0)	0(0)	0(0)	0(0)	0(0)
		10c	c. Indicate the number of independent replications of each experiment, if relevant.	3(0.76)	1(1.39)	0(0)	0(0)	2(1.55)
	Allocating animals to experimental groups	11a	a. Give full details of how animals were allocated to experimental groups, including randomisation or matching if done.	105(26.52)	14(19.44)	18(32.73)	43(30.71)	30(23.26)
		11b	b. Describe the order in which the animals in the different experimental groups were treated and assessed.	395(99.75)	72(100)	55(100)	140(100)	128(99.22)
	Experimental outcomes	12	Clearly define the primary and secondary experimental outcomes assessed (e.g. cell death, molecular markers, behavioural changes).	396(100)	72(100)	55(100)	140(100)	129(100)
	Statistical methods	13a	a. Provide details of the statistical methods used for each analysis.	311(78.54)	56(77.78)	47(85.45)	114(81.43)	94(72.87)
		13b	b. Specify the unit of analysis for each dataset (e.g. single animal, group of animals, single neuron).	111(28.03)	17(23.61)	17(30.91)	35(25)	42(32.56)
		13c	c. Describe any methods used to assess whether the data met the assumptions of the statistical approach.	108(27.27)	24(33.33)	16(29.09)	42(30)	26(20.16)
RESULTS								
	Baseline data	14	For each experimental group, report relevant characteristics and health status of animals (e.g. weight, microbiological status, and drug- or test-naive) prior to treatment or testing (this information can often be tabulated).	1(0.25)	0(0)	0(0)	0 (0)	1(0.78)
	Numbers analysed	15a	a.Report the number of animals in each group included in each analysis. Report absolute numbers (e.g. 10/20, not 50%2).	62(15.66)	13(18.06)	6(10.91)	23(16.43)	20(15.50)
		15b	b. If any animals or data were not included in the analysis, explain why.	18(4.55)	2(2.78)	4(7.27)	5(3.57)	7(5.43)
	Outcomes and estimation	16	Report the results for each analysis carried out, with a measure of precision (e.g. standard error or confidence interval).	360(90.91)	65(90.28)	54(98.18)	130(92.86)	111(86.05)
	Adverse events	17a	a.Give details of all important adverse events in each experimental group.	41(10.35)	2(2.78)	8(14.55)	16(11.43)	15(11.63)
		17b	b. Describe any modifications to the experimental protocols made to reduce adverse events.	0(0)	0(0)	0(0)	0(0)	0(0)
DISCUSSION								
	Interpretation/scientific implications	18a	a. Interpret the results, taking into account the study objectives and hypotheses, current theory and other relevant studies in the literature.	396(100)	72(100)	55(100)	140(100)	129(100)
		18b	b. Comment on the study limitations including any potential sources of bias, any limitations of the animal model, and the imprecision associated with the results2.	29(7.32)	0(0)	3(5.45)	12(8.57)	14(10.85)
		18c	c. Describe any implications of your experimental methods or findings for the replacement, refinement or reduction (the 3Rs) of the use of animals in research	0(0)	0(0)	0(0)	0(0)	0(0)
	Generalisability/ translation	19	Comment on whether, and how, the findings of this study are likely to translate to other species or systems, including any relevance to human biology	108(27.27)	9(12.5)	11(20.00)	25(17.86)	63(48.84)
Funding		20	List all funding sources (including grant number) and the role of the funder(s) in the study.	396(100)	72(100)	55(100)	140(100)	129(100)
Median					18.50	19.00	19.00	20.00
P_25_,P_45_					17.00,20.00	18.00,21.00	18.00, 21.00	18.00, 22.00
Mean Rank					157.99	205.90*	197.26*	219.31*

*:There were statistical differences compared with 2010 Jan.~June.

### Conformity with ARRIVE Guidelines

The ARRIVE checklist scores are shown in Table 2. The range of ARRIVE score is from 12 to 27 with a maximum possible score of 40.The value for each of the median (P25, P45) ARRIVE checklist scores for studies published during January and June2010, July and December 2010, 2011, and 2012 was18.50(17.00,20.00), 19.00 (18.00,21.00), 19.00 (18.00,21.00) and 20.00(18.00,22.00), respectively. Studies published in 2012(*P* = 0.012), 2011 (*P* = 0.015), 2010, July~Dec (*P*<0.017) had a significantly larger ARRIVE checklist score than those published in Jan.~June,2010,respectively.

The least frequently reported items (reported in ≤30% of the studies) were items 3b, 5, 6d, 6f, 7c, 7d, 9b, 9c, 10b, 10c, 11a, 13b, 13c, 14, 15a, 15b, 17a, 17b, 18b, 18c, and 19. These items correspond to information on the scientific background, ethical statements, experimental procedures, sample size, statistical methods, baseline data, numbers of animals analyzed, adverse events, and interpretation/scientific implications. No study provided a time-line diagram or flow chart, information on sample size, information on how the experimental protocol reduced adverse events, or experimental methods or findings for the replacement, refinement, or reduction (the 3Rs) of the use of animals in research. The frequency of reporting the ARRIVE checklist items 7b, 7c, 9a, 9c, 10a, 16, 18b, and 19 differed according to the publication year, but the reporting frequencies for all other individual ARRIVE checklist items were similar for the three years.

## Discussion

Over the last decade, numerous studies have examined the quality of healthcare reporting by assessing the compliance of randomized controlled trials[6],observational studies[7], and systematic reviews[8] with various assessment instruments. The CONSORT statement published in 1996[9], the ARRIVE guidelines[4], and the Gold Standard Publication Checklist (GSPC)[10] published in 2010 represent substantial improvements in methods used for animal studies. Before these guidelines, insufficient reporting occurred in many animal experiments, e.g., a lack of randomization and/or blinding[11–13]. The reporting quality of animal experiments in periodontology, e.g., implant dentistry, published after 2010 has been assessed using the ARRIVE and modified ARRIVE guidelines[2,3,14–17].

In the present study, we focused on 396 reports involving neoplasms in rodents published in Chinese journals between 2010 and 2012. To our knowledge, this is the first assessment of this type of study published in Chinese journals and supported by the NSFC. Although we did not perform an intervention systematic review, we tried to conform to the preferred reporting items for the Preferred Reporting Items for Systematic reviews and Meta-Analyses (PRISMA statement) checklist[8]. In our study, we found that the completeness of these studies regarding the ARRIVE guidelines was suboptimal. The mean ARRIVE checklist score was only 19.48 out of 40, and many studies failed to report important information. Furthermore, half the items in the ARRIVE checklist were reported by <50% of studies. Items 3b, 5, 6d, 6f, 7c, 7d, 9b, 9c, 10b, 10c, 11a, 13b, 13c, 14, 15a, 15b, 17a, 17b, 18b, 18c, and 19 were particularly poorly reported.

All studies included in this analysis were supported by at least one funding organization. Only two studies reported that no conflict of interest existed, and the other studies did not provide a declaration of interests. Accurate reporting of conflicts of interest is very important to enable readers to judge the risk of publication bias.

We found that few studies (1.77%) provided a statement of ethics or provided information on animal welfare. Researchers need to pay close attention to study design, data collection, reporting, and the welfare of the animals to take effective measures to alleviate animal suffering. The ARRIVE guidelines recommend providing a time-line diagram or flow chart of the study design, but neither was part of any study. Similarly, no study described how the sample size was calculated, and therefore it was unclear if the sample sizes were adequate. To minimize the effect of random error on the results, studies should include independent repetitions of each experiment. Although many studies reported the number of repeated measurements, many failed to describe the number of independent replications for each experiment.

As with clinical trials, animal experiments should report if the subjects were randomized into groups and also describe the randomization method in detail to help readers assess the risk of selective bias. Furthermore, it is important to blind outcome assessors and data analysts to the group assignment, thereby reducing the risk of measurement bias for some subjective outcomes. However, blinding was used in only one study.

Although all studies reported the experimental outcomes, many did not define primary and secondary outcomes. Information concerning statistical methods was often incomplete, e.g., the unit of analysis for each dataset was not specified, or the rationale for selecting the statistical approach was not provided.

Only one studies (0.25%) provided the relevant characteristics and health status of the animals, e.g., the strain, sex, age, and weight of the experimental and control animals, which are important factors that allow the reader to judge if the groups were or were not balanced at baseline.

Insufficient information was often provided on the number of animals studied, and no descriptions or explanations were provided concerning animal death or loss to follow-up or incomplete outcome data, making it difficult for readers to evaluate the risk of attrition bias. In addition, although all studies reported the efficacy of the treatment, only 41 (10.35%) reported adverse events, and none described the approach(s) used to reduce adverse events.

Ideally, the discussion section of such studies should address how the results translate to other systems or species and the feasibility of testing the treatment in clinical trials. When animals are used in research, the researcher has the responsibility to work toward the replacement, refinement, and reduction of animal use, i.e., to improve the use value of the animals, reduce the number of animals used, and increase the use of alternative methods. Unfortunately, none of the studies reported information about these issues.

All experimental research has limitations; however, many of the studies did not address this point.

Analysis reveals that the reporting quality of animal experiments reports shows a rising tendency after the first publication of the ARRIVE in June, 2010.However, the quality of reporting was still poor, and future studies should be more transparent and accurately reported. We believe that reporting guidelines, including those of ARRIVE and GSPC, have not been widely used by Chinese researchers who use animals, as our results indicate that neither the ARRIVE nor the GSPC guidelines were used by such researchers from 2010 July to 2012. The ARRIVE and GSPC guidelines outline the minimum information that should be provided when reporting an animal experiment(s). These guidelines should be used when designing, performing, reporting, reviewing, and publishing an animal study. They will also be of use to funding organizations, especially large funding bodies, which have a responsibility to provide financial assistance and to strictly evaluate the quality of the research that is funded[18]. Good reporting allows readers to fully understand the methods and processes involved and to assess the reliability and validity of the findings.

There are several limitations to our study. First, our analyses were limited to studies on rodents, and studies on other animals were excluded. Second, we only included animal experiments involving neoplasms that were published in Chinese journals between 2010 and 2012. Third, our scoring criteria (yes or no) did not allow for partial information. Fourth, we used unweighted scores for the ARRIVE checklist items, although this may not be a valid approach. Finally, in general, the reporting quality of experiments involving neoplasms in animals in Chinese journals may be worse than what we found because all the studies included in our analyses were supported by the NSFC.

## Supporting Information

S1 File The Chinese databases search strategy. (DOC)

S2 File Three hundred and ninety-six animal studies. (DOC)
