# Supplementary material for: Adherence to ARRIVE Guidelines in Chinese Journal Reports on Neoplasms in Animals
Source: PLoS One. 2016 May 16;11(5):e0154657. doi: 10.1371/journal.pone.0154657 (PMC4868299; doi:10.1371/journal.pone.0154657)
Supplement: S1 File — (DOC) [file pone.0154657.s001.doc]

**Text S1 The Chinese databases search strategy.**

**Chinese Science Citation Database (CSCD) search strategy**

**Hyperlink address: http://sdb.csdl.ac.cn/**

#1 SUPERSCRIPTION_CN: Cancer OR TABLOID_CN: Cancer OR KEYWORD_CN: Cancer

#2 SUPERSCRIPTION_CN: Tumor OR TABLOID_CN: Tumor OR KEYWORD_CN: Tumor

#3 SUPERSCRIPTION_CN: Neoplasms OR TABLOID_CN: Neoplasms OR KEYWORD_CN: Neoplasms

#4 #1 OR #2 OR #3

#5 SUPERSCRIPTION_CN: animal experiment OR TABLOID_CN: animal experiment OR KEYWORD_CN: animal experiment

#6 SUPERSCRIPTION_CN: in vivo experiment OR TABLOID_CN: in vivo experiment OR KEYWORD_CN: in vivo experiment

#7 SUPERSCRIPTION_CN: basic research OR TABLOID_CN: basic research OR KEYWORD_CN: basic research

#8 SUPERSCRIPTION_CN: rat OR mice OR TABLOID_CN: rat OR mice OR KEYWORD_CN: rat OR mice

#9 #5 OR #6 OR #7 OR #8

#10 #4 AND #9

#11 FUND_NAMES: National Natural Science Foundation of China

#12 YEAR:[2010 TO 2012]

#13 #9 AND #11 AND #12

**Chinese Journal Full-text Database (CJFD) search strategy**

**Hyperlink address: http://www.cnki.net/**

#1 SU=Cancer

#2 SU=Tumor

#3 SU=Neoplasms

#4 #1 OR #2 OR #3

#5 SU=animal experiment

#6 SU=in vivo experiment

#7 SU=basic research

#8 #9 OR #10 OR #11

#9 FU=National Natural Science Foundation of China

#10 YE=(2010+2011+2012)

#11 #4 AND #8 AND #9 AND #10
