# Supplementary material for: Adherence to ARRIVE Guidelines in Chinese Journal Reports on Neoplasms in Animals
Source: PLoS One. 2016 May 16;11(5):e0154657. doi: 10.1371/journal.pone.0154657 (PMC4868299; doi:10.1371/journal.pone.0154657)
Supplement: S2 File — (DOC) [file pone.0154657.s002.doc]

***Text S2 Three hundred and ninety six included studies***

1. Cai QY, Wang BL, Jin J, Chen JP, Ye SL, Ying AM, Wan XJ, Wei J (2012) Anti-tumor effect of the antigen specific CTLs induced by dendritic cells transfected with total RNA of lung adenocarcinoma cell in vivo. *Chinese Journal of Blood Transfusion* 25(4):312-317
2. Cai Y, Zhou W, Zhou SJ, Tang Y, Gong JP, Liu CA, Li SW (2011) All-transretinoic acid enhances killing effect of ultrasound microbubble carrying herpes simplex virus thymidine kinase on hepatocellular carcinoma transplanted nude mice. *Journal of Third Military Medical University* 30(20):2112-2115
3. Cao LH, Zhou JH, Lin WQ, Tan HY, Zeng WA, Zeng YX (2010) Effect of morphine on suppression of cisplatin on tumor growth of human nasopharyngeal carcinoma CNE-2 xenografts in nude mice. *Journal of Sun Yat-Sen University(Medical Sciences)* 31(3):371-375
4. Cao FC, Han YL, Chen B, Liu H, Ding H, Huang MX, Ye Y (2011) Effect of eupolyphaga fibrinolytic protein on microvessel density and the expression of vascular endothelial growth factor in mice tumor. *Journal of Chinese Medicinal Materials* 34(5):676-679
5. Chai W, He XJ, Zhu JX, Lv C, Zhao HY, Lv AP, Yu CY (2012) Immunoregulatory effects of astragalus polysaccharides on myeloid derived suppressor cells in B16-F10 tumor bearing mice. *Chinese Journal of Basic Medicine in Traditional Chinese Medicine* 18(1):63-65
6. Chen DZ, Tang QS, Xiang JY, Xu F, Zhang L, Wang JF (2011) Combined anti-tumor therapeutic effect of targeted gene, hyperthermia, radionuclide brachytherapy in breast carcinoma. *Chinese Journal of Nuclear Medicine* 31(2):82-86
7. Chen HB, Shen B, Li L, Cheng HB, Zhou HG, Wang YM, Wu MH (2011) Xiaoai Jiedu Recipe inhibits transplanted hepatocarcinoma H22 tumors and the related mechanism. *Chinese Journal of Cancer Biotherapy* 18(1):28-32
8. Chen J, Lin AH, Hou T, Chen ZP, Cai BC (2012) Effect of lipid composition on antitumor activity of stealth liposomes containing total alkaloids from seeds of Strychnos nux-vomica L. *Chinese Traditional Patent Medicine* 34(2):238-241
9. Chen J, Wang LN, Zhang YB, Yang JP, Xu QN, Wang XY, Zuo JL 2010 Role of P2Y1 purinergic receptors in the spinal cord in a rat model of bone cancer pain. *Chinese Journal of* *Anesthesjology* 30(10):1220-1223
10. Chen JY, Geng L, Zhang XQ, Yang TT, Li HW, He X, Pu XF, Peng F (2012) Effect of periplaneta Americana extract CⅡ-3 on angiogenesis in H22 hepatoma-bearing mice. *Journal of Chinese Oncology* 18(4):274-276
11. Chen ML, Chen J, Hou T, Fang Y, Sun WW, Hu RR, Cai BC (2011) Effect of phospholipid composition on pharmaceutical properties and anti-tumor activity of stealth liposomes containing brucine. *China Journal of Chinese Materia Medica* 36(7):864-867
12. Chen SS, Liu XJ, Cao F, Xiao XP, Tian XF, Tian YK (2010) Analgesic effects of ropivacaine by intracerebral injection in a rat model of bone cancer pain. *Herald of Medicine* 40(9):425-430
13. Chen TC, Wei PK (2011) Proteomic study of xiaotansanjie recipe intervened human gastric tumor-bearing mice. *Chinese Journal of Information on Traditional Chinese Medicine* 18(1):44-46
14. Chen TC, Wei PK, Jia ZM, Wei Z (2012) Comparative serum proteomic study of xiaotan sanjie recipe intervened MKN-45 human gastric tumor-bearing nude mice. *Chinese Journal of Information on Traditional Chinese Medicine* 9(8):44-47
15. Chen X, Yang GM, He YY, Cai BC (2011) Antitumor effect of alkaloids from Oxytropis falcata on S180 bearing mice. *China Journal of Traditional Chinese Medicine and Pharmacy* 26(11):2540-2542
16. Chen XF, Liu Q, Liu S, Zhang XF, Yang SF (2011) Influences of coupled medicinal-Shechuangzi (Fructus Cnidii) and Buguzhi(Fructus Psoraleae)on survival time and bone injury in nude mice with breast cancer with bone metastasis. *Journal of Beijing University of Traditional Chinese Medicine* 35(5):317-322
17. Chen XF, Liu S, Jin H, Zhang XF, Liu Q (2012) Study on characteristics of absorption into blood of common cnidium fruit-malaytea scurfpea fruit in breast cancer bone metastasis nude mice model. *Chinese Journal of Experimental Traditional Medical Formulae* 18(6):175-180
18. Chen XF, Liu S, Yang SF, Liu Q (2011) Dose-effect relationship of Chinese medicines cnidium fruit-psoralea fruit in pairs on the body weight and bone metabolism of mammary cancer bone metastasis in nude mice. *Journal of Traditional Chinese Medicine* 52(24):2128-2134
19. Chu SH, Ma YB, Feng DF, Zhang H, Qiu JH, Zhu ZA (2011) 5-Aza-2’-deoxycytidine-induced up-regulation of SLC22A18 expression in U251 cells gliomas and its inhibitory effects on the gliomas growth in nude mice. *Chinese Journal of Clinical Neurosurgery* 16(12):732-734
20. Cui XY, Zhu SJ, Cui LX, Qin XF, Yang L, Wang P (2011) Sonodynamic therapy with chlorin e6 for Ehrlich ascites tumor-bearing mice. *Acta Laboratorium Animalis Scientia Sinica* 19(6):451-455
21. Deng B, Jia LQ, Gao FY, Cui J, Li H, Li H (2010) Affect of Bushen Zhuanggu Fangyao upon Destruction and Density of Bone in Osteocarcinoma Transfer. *Jilin Journal of Traditional Chinese Medicine* 30(1):72-73
22. Deng LC, Xu SY, MaoSH, Yi C, Huang Y (2011) Anti-tumor effect of Bifidobacterium infantis-mediated sFlt-1 eukaryotic expression system on Lewis lung cancer in mice. *West China Journal of Pharmaceutical Sciences* 26(6):564-566
23. Deng N, Gao H, Guo YF, Qiu XF, Feng J, Zheng P, Dai GD (2012) Preliminary study on inhibitory effect of matrine on bladdercancer via regulation of cyclooxygenase-2 expression in rats. *Chinese Pharmacological Bulletin* 28(3):375-378
24. Dong BS, Xie RL, Pang H, Bian JJ, Guo WH, Cheng Y, Li XY (2010) Effects of AMD3100 on progression of Lewis lung carcinoma and accumulation of myeloid derived suppressor cells in tumor-bearing mice’s spleen. *Acta Academiae Medicinae Militaris Tertiae* 32(12):1301-1304
25. Feng JF, Xu B, Ge HY, Liu XH (2011) Oncolytic adenovirus expressing lipocalin 2 suppresses the growth of transplantation tumors of colonic carcinoma. *Chinese Journal of General Surgery* 26(5):363-366
26. Feng QM, Wu X, Liu P, Peng M, Wang Y, Di W (2011) Synergistic antitumor activity of chemotherapy and immunotherapy in the treatment of established rat solid tumors. *Chinese Journal of Microbiology and Immunology* 31(7):632-637
27. Feng ST, Li H, Sun CH, Cai HS, Zhou J, Shuai XT, Li ZP, Meng SF (2011) The MRI study of supraparamagnetic ironic oxide loaded polymeric nano-vesicles in human colonic carcinoma xenograft in nude mice. *Chinese Journal of Radiology* 45(3):288-292
28. Feng XQ, Wang JH, Xu XN, Zhang B, Wang SJ, Liu HS, Lin N (2010) Anti-glioma effect of combination of bFGF-siRNA and Vpr in nude mice. *Chinese Journal of Oncology* 32(10):725-728
29. Fu DL, Zhong T, Li HC, Zhang P, Gan WM, Wang L (2011) Inhibitive effect of matrine on the growth of subcutaneous implanted tumor model of ACHN renal cell carcinoma in nude mice. *Journal of Modern Urology* 16(1):10-13
30. Fu YW, Pan YL, Qin L, Sun L, Liu YM (2011) Inhibition of Ang-2 and RGS-5 expression by nanogold results in normalization of vascellum in hepatic tumor. *Chinese Journal of Pathophysiology* 27(12):2247-2252
31. Gao J, Li JS, Xu GL, Lai WD, Ma JL, Yu JH, Ge YS (2010) Effects of celecoxib combined with fiuvastatin on tumor growth and cell apoptosis in a xenograft model of hepatocellar carcinoma. *Chinese Journal of Hepatology* 18(12):900-904
32. Gao J, Gao GL, Yu XL, Zhang YY, Wang F (2010) Expression and Significance of P53 and VEGF in Serum and Xenograft of SKOV3 Tumor-Bearing Nude Mouse Model of Adverse Psychological Stress. *Tianjin Medical Journal* 38(10):1072-1075
33. Gao J, Gao GL, Yu XL, Zhang YY, Wang F (2010) Effect of chronic psychological stress on serum NE,IL-10 and CA125 Levels in SKOV3 tumor nude mice. *Progress in Obstetrics and Gynecology* 19(8):589-592
34. Gao J, Cheng Y, Kong J, Ke S, Ding XM, Sun WB (2011) Inhibitory effect and mechanism of fluorouracil implants on residual tumor of mouse H22 model following radiofrequency ablation. *Chinese Journal of Experimental Surgery* 28(8):1299-1302
35. Gao QL Li HB, Yan YM, Chen YQ, Yang F (2012) The relation of inhibiting angiogenesis and inducing cell apoptosis of melittin(Mel) on xenotransplanted models of nude mice. *Fudan University Journal of Medical Sciences* 39(3):283-288
36. Gao QL, Yang F, Yao YM, Wang HZ, Liu HM, Chen YQ (2011) Experimental Study of Treatment and Safety of Melittin on Xenotransplanted Models of Nude Mice. *Liaoning Journal of Traditional Chinese Medicine* 38(7):1300-1303
37. Gao QL, Yao YM, Yang F, Tian TD, Chen YQ (2011) Experimental Study of the Relation of Inhibiting Vasculogenic Mimicry and Inducing Cell Apoptosis of Melittin on Osteosarcoma Xenotransplanted Models of Nude Mice. *China Journal of Chinese Medicine* 26(9):1027-1030
38. Gao Q, Gu XP, Zhang J, Zhou XF, Wang JH, Ma ZL (2010) Role of spinal cord TNF-α in the development of bone cancer pain in mice. *Chinese Journal of Anesthesiology* 30(3):331-333
39. Gong YQ, Wen B (2010) Effect of Zuojin Pill on Expression of APC and Activity of DNA Methyltransferase in Colorectal Adenomas. *Journal of Nanchang University*(Medical Science) 50(12):46-49
40. Gu LH, Liu H, Wei JE, Yu H, Lu XD, Chen GH, Zhou RX (2010) Effects of melatonin on surviving expression in mice with gastric cancer. *Chinese Journal of Histochemistry and Cytochemistry* 19(6):583-586
41. Guo QS, Huang X, Li SL (2011) Effect of peroxiredoxin I gene silencing on the radiosensitivity of breast carcinoma MCF-7 cell xenograft in nude mice. *Journal of Southern Medical University* 31(7):1119-1123
42. Guo W, Wei PK, Gui MW (2010) Effect of Xiaotan Sanjie Recipe on E-cad Methylation in Nude Mice with Orthotopically Transplanted Human Gastric Cancer. *Journal of New Chinese Medicine* 51(12):113-115
43. Guo W, Wei PK, Gui MW, Qin ZF (2010) Effect of Xiaotansanjiefang on DNA Methylation of P16 in Nude Mouse Model of Human Gastric Carcinoma Cells Using Orthotopic Transplantation. *Chinese Journal of Information on Traditional Chinese Medicine* 17(10):28-31
44. Guo WH, Bian JJ, Dong BS, Zhang YF, Li XY (2010) Effect of Angiogenesis on Growth and Metastasis of Lewis Lung Carcinoma. *Cancer Research on Prevention and Treatment* 37(4):378-381
45. Guo XY, Si TG, Guo Z, Wang HT (2010) Experimental study of anti-tumor immunologic response induced by cryoablation for prostate cancer. *National Medical Journal of China* 90(14): 952-955
46. Hai LD, Ma KX, Huang L, Niu WC, Wang RZ (2012) Cetuximab and the antitumor effect of different radiation pattern in CNE-2 Xenografted and the expression of Bax,Bcl-2. *Journal of Xinjiang Medical University* 35(3):276-281,287
47. He D, Ge W, Li CH, Zhao ZY, Xu XM, Yang F (2010) Effects of Rh-endostar in Combination with Radiotherapy on Rats with Lung Cancer. *Chinese Journal of Lung Cancer* 13(04):386-390
48. He WF, He DW, Ma C, Zhao D, Zhang YB, Bian ZL (2011) Gallic acid inhibits growth of human neuroblastoma xenograft in mice. *Acta Academiae Medicinae Militaris Tertiae* 33(2):107-110
49. He XD, Gong P, Qi CL, He W, Wang LJ, Li WD (2011) The anti-tumor effects of andrographolide drop pills on murine B16 melanoma. *Journal of Guangdong Pharmaceutical College* 27(2):163-165
50. He XJ, Jia RP, Shao GQ, Xu LW, Wang ZZ, Huang PL, Wu JP, Wang J (2010) Implantation brachytherapy with 32P-chromic phosphate-poly (L-lactide) delayed-release particles for prostate cancer in nude mice. *National Journal of Andrology* 16(10):872-876
51. He XJ, Jia RP, Wang ZZ, Xu LW, Shao GQ, Huang PL, Cao P (2010) 32P-chromic phosphate-poly (L-lactide) implantation brachytherapy on prostatic carcinoma with lymphatic metastasis. *Chinese Journal of Experimental Surgery* 27(8):1144-1147
52. Hu HZ, Wang WG, Ma JM, Su CQ, Jiang Y (2012) Adenovirus E1a gene enhances P16 gene-induced apoptosis of hepatocellular carcinoma SMMC-7721 cells. *Chinese Journal of Cancer Biotherapy* 18(6):630-634
53. Hu JH, Yang JP, Liu L, Li CF, Yao M (2010) Changes in hanges in expression of phosphorylated cyclic AMP response element binding protein (pCREB) in dorsal horn of sinal cord in a rat model of bone cancer pian. *Chinese Journal of Pain Medicine* 16(5):285-288
54. Huang J, Li SM, Zhu Q, Cao HX, Zhang YP (2012) The effect of silent homo sapiens eukaryotic translation elongation factor 1 alpha 2 gene on the growth of pancreatic cancer xenograft in nude mice. *Chinese Journal of Digestion* 32(2):98-102
55. Huang LZ, Wang CX, Ma RD, Yu LJ, Su WM, Liao MN, Yu YX (2010) Effects of Intraperitoneal Injection of Fuantai-03 on Metastasis-related Genes and Metastasis in Mouse Lewis Lung Carcinoma and B16 Melanoma. *Chinese Journal of Cell Biology* 32(2):223-228
56. Huang SX, Xie MQ, Li Y, Yuan XY, Chen SJ (2011) An Experimental Study of the Distribution and Targeting Effect of CDDP-loaded Magnetic Nanoparticles in Nude Mice with Transplanted Nasopharyngeal Carcinoma. *Journal of Medical Research* 40(2):22-26
57. Huang X, Huang GX, Song HZ, Chen YT, Chen LB (2011) Cisplatin pretreatment enhances the antitumor activity of cytokine-induced killer cells in a T cell-dependent manner. *Oncology Progress* 9(6):639-645
58. Huang XM, Lian L, Zhou YF, He XS, Wu XJ, Tan WY, Lan P (2012) Correlation of IL-10 and TGF-β1 expression with the liver metastasis of colon carcinoma in mice. *Chinese Archives of General Surgery(Electronic Edition)* 6(3):193-199
59. Ji MS, Huang ZL, Li YJ, Huang SF, Ceng JM, Liu DB, Cao WX, Feng WL (2011) Curative Effect of PTD-OD-HA Fusion Protein on Subcutaneous Solid Tumor Caused by K562 Cells in Nude BALB/c Mice. *Chinese Journal of Biologicals* 24(8):945-947
60. Jia XM, Yang JP, Wang LN, Zhong LM, Gao JL, Xu QN, Wang XY, Zuo JL (2010) Mechanism and expression of KCC2 in the spinal cord of bone cancer pain rats. *Chinese Pharmacological Bulletin* 26(11):1463-1466
61. Jang X, Sun XY, Wang L, Sun L, Jang HC, Qiao HQ (2010)Experimental study on combination of meloxicam and anti-angiogenic therapy in liver cancer.[*Journal of Harbin Medical University*](http://202.201.7.11/kns55/loginid.aspx?uid=M1JwS1Y0akdIUkU0U3NlY3R5S1dHUWM3L1I2ZjdVQVJneTRFUC9nTVYxUW9nV3c3&p=Navi/Bridge.aspx?LinkType=BaseLink&DBCode=cjfq&TableName=CJFQbaseinfo&Field=BaseID&Value=HYDX)44(3):208-211
62. Jang P, Qian Y, Feng AP, Chen SY, Chu SJ, Zhang L, Wu Y, Zhang N, Luo Q (2010)Inhibitory effect of live-attenuated Listeria monocytogenes-based vaccines carrying mare-1 gene on mouse malignant melanoma.*CHINESE JOURNAL OF DERMATOLOGY* 43(7):455-459
63. Jin AH, Piao L, Yin XZ, Quan JS (2012)Anti-tumor effect of iridoid glucosides from Boschniakia rossica in H22-bearing mice.[*Chinese Traditional and Herbal Drugs*](http://202.201.7.11/kns55/loginid.aspx?uid=M1JwS1Y0akdIUkU0U3NlY3R5S1dHUWM3L1I2ZjdVQVJneTRFUC9nTVYxUW9nV3c3&p=Navi/Bridge.aspx?LinkType=BaseLink&DBCode=cjfq&TableName=CJFQbaseinfo&Field=BaseID&Value=ZCYO)43(2): 332-335
64. Jin QK, Xu JH, Xiao HJ, Lu H, Yu WY, Wang GJ, Sun J, Fan ZZ (2012)Changweiqing decoction increase the effects of oxaliplatin in human colorectal cancer cell line HCT.[*Global Traditional Chinese Medicine*](http://202.201.7.11/kns55/loginid.aspx?uid=M1JwS1Y0akdIUkU0U3NlY3R5S1dHUWM3L1I2ZjdVQVJneTRFUC9nTVYxUW9nV3c3&p=Navi/Bridge.aspx?LinkType=BaseLink&DBCode=cjfq&TableName=CJFQbaseinfo&Field=BaseID&Value=HQZY)5(3):175-179
65. JU R, Wu DW, Guo L, Li J, Ye CY, Zhang DC (2011)Carboxyamidotriazole and low dose dexamethasone significantly inhibit the growth of A549 xenograft tumors.[*Chinese Medicinal Biotechnology*](http://202.201.7.11/kns55/loginid.aspx?uid=M1JwS1Y0akdIUkU0U3NlY3R5S1dHUWM3L1I2ZjdVQVJneTRFUC9nTVYxUW9nV3c3&p=Navi/Bridge.aspx?LinkType=BaseLink&DBCode=cjfq&TableName=CJFQbaseinfo&Field=BaseID&Value=ZYSW)4(6): 101-104
66. Ju DW, Wei PK, Lin HM, Sun DZ, Yu S,Xiu LJ (2010).Effects of xiaotan sanjie decoction on expressions of interleukin-8 and its receptors in gastric tumor xenografts and gastric tissue adjacent to the tumor in mice.[*Journal of Chinese Integrative Medicine*](http://202.201.7.11/kns55/loginid.aspx?uid=M1VoQ2taNFZpbDhWZ21PZlN4ak5LejBmNE9kTDhvS2srckltMEUrQnd0SlFSVml6&p=Navi/Bridge.aspx?LinkType=BaseLink&DBCode=cjfq&TableName=CJFQbaseinfo&Field=BaseID&Value=XBZX)8(1): 74-79
67. 149.Kong FB, Wang XT, Xie YB, Xiao Q (2012)Effects of the recombinant retroviral vector with Cdx2 on growth of subcutaneous tumor of human gastric cancer in nude mice.[*China Journal of Modern Medicine*](http://202.201.7.11/kns55/loginid.aspx?uid=M1VoQ2taNFZpbDhWZ21PZlN4ak5LejBmNE9kTDhvS2srckltMEUrQnd0SlFSVml6&p=Navi/Bridge.aspx?LinkType=BaseLink&DBCode=cjfq&TableName=CJFQbaseinfo&Field=BaseID&Value=ZXDY)22(4):6-10
68. Kong GM, Zhang XR, Wu KY, Zhao FY, Zhu HH, Zhang XD, Liao YX, Pu p (2012) Inhibitory effect of KDRspecific monoclonal antibody on tumor growth in nude miceI bearing human gastric cancer.[*World Chinese Journal of Digestology*](http://202.201.7.11/kns55/loginid.aspx?uid=M1VoQ2taNFZpbDhWZ21PZlN4ak5LejBmNE9kTDhvS2srckltMEUrQnd0SlFSVml6&p=Navi/Bridge.aspx?LinkType=BaseLink&DBCode=cjfq&TableName=CJFQbaseinfo&Field=BaseID&Value=XXHB).19(28):2926-2930
69. Sun M, Zeng CC, Xiong HL, Liu HP, Liu SH (2011)Effect of Gold Nanoparticles on Tissue Optical Properties of Gastric Tumor Tissue in Near-Infrared Spectral in Nude Mice*.*[*Acta Optica Sinica*](http://202.201.7.11/kns55/loginid.aspx?uid=ajcvcElPcUc3K2gvelBxL1FaeDJoNjZkMUxrTUtCdnhacFQ0RFcxODh3K1NZWjg3&p=Navi/Bridge.aspx?LinkType=BaseLink&DBCode=cjfq&TableName=CJFQbaseinfo&Field=BaseID&Value=GXXB).31(03):1-5
70. Sun R, Sun B, Wang SJ, Pan SH, Wang G, Cheng H, Xue DP ,Jang HC(2010)An experimental study of gemcitabine inducing pancreatic cancer cell apoptosis potentiated by nuclear factor-kappa B P65 siRNA.Chinese journal of surgery .48(2):128-13
71. Li Y, Wang XP, Wu YF, Huang WG, Huang K, Huang SL, Wei Q, Zhou DH, Fang JP, Xue HM (2011)Influences of bone marrow mesenchymal stem cells infusion on anti-tumor activities of cytokine-induced killer/natural killer cells from umbilical cord blood in K562 NOD/SCID mice*.Chinese Journal of Organ Transplantation*.48(2):128-13
72. Li BL, Zhang GX, Hou XL, Gong DJ, Yuan Y, Liu XH, Huang SD, Xu ZY (2011)Suppression of vascular endothelial growth factor and angiopoietin-2 to treat adenocarcinoma of lung.*Chinese journal of clinicians.*5(13):3756-376
73. Li B, Zhu XM, Gao MH (2011)Treatment of Hela Tumor in mice with C-phycocyanin Mediated Photodynamic Therapy and Its Immune Mechanism Underlying Apoptosis.*Chinese journal of laser medicine & surgery .*(01):1-6
74. Li CF, Yang JP, Wang LN, Liu L, Hu JH, Liu SL (2011)The attenuate hyperalgesia effect of intrathecal u0126 in a rat model of bone cancer pain.*Chinese pharmacological bulletin*.27(07):949-951
75. Li CY, Ge W, Zhen YF, Xu XM (2011)Effect of endostar combined with radiotherapy on the expression of HIF-1α, AQP1 and VEGF in the mice with lung caner.[*Biomedical engineering and clinical medicine.*](http://c.wanfangdata.com.cn/Periodical-swyxgcylc.aspx)15(04):370-373
76. Li DT, Sun GZ, Wu ZK, Li JL, Xin X, Chen YY, Pei YX, Qi X( 2012)Effect of Yiqihuoxue,Ruanjianjiedu recipe on expression of apoptosis regulate gene in mice with H22615 tumor.[*Chinese Journal of Integrated Traditional and Western Medicine on Liver Diseases*](http://202.201.7.11/kns55/loginid.aspx?uid=alNBV1VHb3VGMjVmd3Z5OU1McXhiT2pZd28zZ092dWlQeWFPcWpxTndjWkpQb2Ru&p=Navi/Bridge.aspx?LinkType=BaseLink&DBCode=cjfq&TableName=CJFQbaseinfo&Field=BaseID&Value=ZXGB)*.*25（4):347-349
77. Li DH, Xie XL, Zhang YL, Geng JG, Bai X, Zhang Y (2010)Study on gene expression profile of P53 signaling pathway regulated and controlled by traditional Chinese medicine in rats model of hysteromyoma.[*Maternal and Child Health Care of China*](http://202.201.7.11/kns55/loginid.aspx?uid=alNBV1VHb3VGMjVmd3Z5OU1McXhiT2pZd28zZ092dWlQeWFPcWpxTndjWkpQb2Ru&p=Navi/Bridge.aspx?LinkType=BaseLink&DBCode=cjfq&TableName=CJFQbaseinfo&Field=BaseID&Value=ZFYB)*.*20（6):4910-4913
78. Li DX, Wu XH, Zhang L, Wang L (2010)Treatment effect of chronic lentivirus mediated mesothelin siRNA on xenograft tumor of ovarian carcinoma.[*Chinese Journal of Cancer Biotherapy*](http://202.201.7.11/kns55/loginid.aspx?uid=alNBV1VHb3VGMjVmd3Z5OU1McXhiT2pZd28zZ092dWlQeWFPcWpxTndjWkpQb2Ru&p=Navi/Bridge.aspx?LinkType=BaseLink&DBCode=cjfq&TableName=CJFQbaseinfo&Field=BaseID&Value=ZLSW)25（33):455-457
79. Li GQ, Li XL, Xie BB, Chen YH, Lei HW, Zou LJ (2010)Study on the radiosensitivity of nude mice model transplanted lung adenocarcinoma cell treated by β-elemene .*Chinese Journal of Radiological Medicine and Protection*17（4):687-691
80. Li H, Cao JG (2011)Recombinant vascular basement-membrane-derived multifunctional peptide inhibits the growth and angiogenesis of lung carcinoma in nude mouse xenograft model.*Journal of Hunan Normal University(Medical Science)*38（6):9-10
81. Li H, Cao JG (2011)Recombinant vascular basement-membrane-derived multifunctional peptide inhibits the growth and angiogenesis of lung carcinoma in nude mouse xenograft model.*Journal of Hunan Normal University(Medical Science)*38（6):9-10
82. Li H, Wang JF, Zhang X, Liu D, Xue CH (2011)Inhibitory effect of chondroitin sulfate isolated from Isostichopus badionotus on experimental lung metastasis of mouse melanoma cell line.*Chinese Pharmacological bulletin*8（1）:623-628
83. Li J, Cai HD, Yang MJ, Hao J .(2011)Ad-hDCT (tumor vaccine) inhibiting the proliferation of intracranial B16 melanoma cells in C57BL/6 mice.*Acta anatomica sinica .*27（5）:787-791
84. Li J, Hao Y, Zhang YL, Xie W, Li J (2011)Berberine inhibits tumor-associated macrophages in subcutaneous tumor of mice.*Chinese Journal of Histochemistry and cytochemistry*42（6）:203-206
85. Li JQ, Yu JM, Song XR, Liu W (2011)Effect of HIF-1α siliencing on human lung cancer xenografts radiosensitivity in nude mice.*Chinese Journal of Cancer Prevention and Treatmen*20（3）:1665-1667
86. Li JX, Zhang QH, Hu XX, Qi C(2012)Effects of "Zengmian Yiliu Decoction" on expression of resistance related genes HIF-1α,Glut1,MDR1,P-gp in nude mice with Cisplatin-resistant ovarian cancer.[*Shanghai Journal of Traditional Chinese Medicine*](http://202.201.7.11/kns55/loginid.aspx?uid=aHladmdpNVRuS0dleGJ6RmMvaWRGRC8xclpDTDc1V3VrMjNhQ3BORFRMdkRJbGdG&p=Navi/Bridge.aspx?LinkType=BaseLink&DBCode=cjfq&TableName=CJFQbaseinfo&Field=BaseID&Value=SHZZ).18（21）:61-6
87. Li L, Shen B, Chen HB, Wang YC, Song Q, Wu MH (2011)Effects of "Zengmian Yiliu Decoction" on expression of resistance related genes HIF-1α,Glut1,MDR1,P-gp in nude mice with Cisplatin-resistant ovarian cancer.[*Liaoning Journal of Traditional Chinese Medicine*](http://202.201.7.11/kns55/loginid.aspx?uid=aHladmdpNVRuS0dleGJ6RmMvaWRGRC8xclpDTDc1V3VrMjNhQ3BORFRMdkRJbGdG&p=Navi/Bridge.aspx?LinkType=BaseLink&DBCode=cjfq&TableName=CJFQbaseinfo&Field=BaseID&Value=LNZY)46（1）:2283-2284
88. Li M, Song Y (2011)Study on the inhibitory effect of inositol hexaphosphate on the growth of subcutaneous transplanted tumor of ht-29 cell in nude mice. ,[*Acta Nutrimenta Sinica*](http://202.201.7.11/kns55/loginid.aspx?uid=aHladmdpNVRuS0dleGJ6RmMvaWRGRC8xclpDTDc1V3VrMjNhQ3BORFRMdkRJbGdG&p=Navi/Bridge.aspx?LinkType=BaseLink&DBCode=cjfq&TableName=CJFQbaseinfo&Field=BaseID&Value=YYXX)38（11):121-125
89. Li MH, Chen SJ, Shao ZH, Zhang W, Jiang H, Wang PJ (2010)Study of bone mesenchymal stem cells tropism for hepatic tumors and effect on the form of tumor stromal.*NATIONAL National medical journal of china* 32（2):349-354
90. Li Q, Liu NN, Zhao CG, Zhao N, Wang Y, Sun Y, Fan ZZ (2010)Establishment of a mouse model of chronic Helicobacter pylori infection-induced gastric adenocarcinoma and investigation of the effect of Helicobacter pylori infection on angiogenesis.[*World Chinese Journal of Digestology*](http://202.201.7.11/kns55/loginid.aspx?uid=&p=Navi/Bridge.aspx?LinkType=BaseLink&DBCode=cjfq&TableName=CJFQbaseinfo&Field=BaseID&Value=XXHB)90（5）:1637-1642
91. Li Q, Liu S, Yang SF (2010)Inhibition of Bone Metastasis of Breast Cancer by Fructus Psoraleae and Fructus Cnidii in vivo.[*Acta Universitatis Traditionis Medicalis Sinensis Pharmacologiaeque Shanghai*](http://202.201.7.11/kns55/loginid.aspx?uid=&p=Navi/Bridge.aspx?LinkType=BaseLink&DBCode=cjfq&TableName=CJFQbaseinfo&Field=BaseID&Value=SHZD)18（16）:53-58
92. Li RW, Wei W, Liu LC, Wen DS, Chen X (2010)The biological behaviour influence of Anti-sense miRNA-21/ rAV-Tumstatin viral vector on nude mice bladder cancer.[*Chinese Journal of Laboratory Diagnosis*](http://202.201.7.11/kns55/loginid.aspx?uid=&p=Navi/Bridge.aspx?LinkType=BaseLink&DBCode=cjfq&TableName=CJFQbaseinfo&Field=BaseID&Value=ZSZD)24（1）:782-784
93. Li SW, Xue H,Wu J,Hu XF,Wang JJ.Characteristics of Activity Rhythm of Mice Bearing Breast Tumor by Electroacupuncture on Changqiang(GV 1) and Baihui(GV 20).[*Journal of Chengdu University of Traditional Chinese Medicine*](http://202.201.7.11/kns55/loginid.aspx?uid=&p=Navi/Bridge.aspx?LinkType=BaseLink&DBCode=cjfq&TableName=CJFQbaseinfo&Field=BaseID&Value=CDZY)16（5）:9-13
94. Li SJ, Luo G, Pang H, Li SL(2010)Growth inhibition and biodistribution of phage display recombinant antibodies against lung adenocarcinoma cell line overexpressing prx I and the therapeutic effect of 131I-scfv on transplanted tumors in nude mice.*Journal of China Medical University*35（1）:790-793
95. Li T, Zeng ZC, Wang L, Qiu SJ, Zhi XL, Zhou JW, Yu HH, Tang YQ (2011)The role of TMPRSS4 in radiation induced metastasis of hepatocellular carcinoma.*Chin J Hepatobiliary Surg*17（12）:1009-1012
96. Li T, Tang YQ, Zhou JW, Fan J, Hu SY, Zhi XT(2012)The mechanism of pseudomonasaeruginosa mannose sensitive hamemagglutination vaccine in inducing apoptosis of hepatocellular carcinoma*.Chinese Journal of Hepatobiliary Surgery*17（12）:1009-1012
97. Li T, Zhi XT, Zhou JW, Dong ZR, Qu H, Yu HH, Wang L, Tang YQ(2011)The role and mechanism of low-dose aspirin with IFN-α in inhibiting growth and metastasis of hepatocellular carcinoma.*Chinese Journal of Hepatobiliary Surgery*10（17）:50-53
98. Li T, Yang YB, Meng LR, Li XM, Xu CF(2012)Bioluminescence imaging evaluation of the inhibitory effect of rapamycin in nude mice bearing endometrial cancer cell lines.[*Chinese Journal of Cancer Prevention and Treatment*](http://202.201.7.11/kns55/loginid.aspx?uid=MnRPdkZyVVNucjIwdU5GUEtJSi9mME4wOVcrUDNtUmR0RVJ5ZUt0Tm5rTWQ3Q1JR&p=Navi/Bridge.aspx?LinkType=BaseLink&DBCode=cjfq&TableName=CJFQbaseinfo&Field=BaseID&Value=QLZL)18（1）:1221-1224
99. Li W, Yang JP, Wang LN, Ren CG, Zhou J (2011)Effectsof intrathecal injectionof baclofenonmechanical hyperalgaof mirror imagepain in rats.[*Medical Journal of National Defending Forces in Southwest China*](http://202.201.7.11/kns55/loginid.aspx?uid=MnRPdkZyVVNucjIwdU5GUEtJSi9mME4wOVcrUDNtUmR0RVJ5ZUt0Tm5rTWQ3Q1JR&p=Navi/Bridge.aspx?LinkType=BaseLink&DBCode=cjfq&TableName=CJFQbaseinfo&Field=BaseID&Value=XNGF)18（16）:1188-1190
100. Li XW, Jing XZ, Zhang XW (2011)Experimental study of TTF1 regulating ERK signal transduction pathway*Yanbian University*12（34）:249-252
101. Li XW, Jing XZ, Zhang XW (2011)Experimental study of TTF1 regulating ERK signal transduction pathway*Yanbian University*12（34）:249-252
102. Li XW, Jing XZ, Zhang XW (2011)Inhibition of tumor angiogenesis by TTF1[Journal of *Medical Science Yanbian University*](http://202.201.7.11/kns55/loginid.aspx?uid=MnRPdkZyVVNucjIwdU5GUEtJSi9mME4wOVcrUDNtUmR0RVJ5ZUt0Tm5rTWQ3Q1JR&p=Navi/Bridge.aspx?LinkType=BaseLink&DBCode=cjfq&TableName=CJFQbaseinfo&Field=BaseID&Value=YBYB)23（9）:249-252
103. Li XT, Zhao L, Chen F (2012)Preparation of Vinorel bine Liposomes and Their Tissue Distribution in Mice.*China Pharmacy*34（4）:788-790
104. Li YX, Cao JN, Qi LH, Dong JX, Chi XC, Xu J(2010)Gemcitabine (Gemzer) up-regulatesThe expression Of gene tusc2 and lats2 in breast cancermice*.ACTA ANATOMICA SINICA* 36（10）:832-836
105. Li Z, Liu YH, Xue YX, Liu LB (2010)Mechanism for endothelial monocyte activating polypeptide-II-induced increase in blood-tumor barrier permeability.[*Progress of Anatomical Sciences*](http://202.201.7.11/kns55/loginid.aspx?uid=aDNKRDFBMmdLKzcwRkZiVHNzT3crL3NjajF0MU1EZHBHeTdHM2cwdTA0UHhTZ0p2&p=Navi/Bridge.aspx?LinkType=BaseLink&DBCode=cjfq&TableName=CJFQbaseinfo&Field=BaseID&Value=JPKX)41（6）:33-36
106. Lei Z, Qu GT, Li XM, Lu XY, Zhang JN (2011)A study of enhancement of radiosensitivity of the allograft model of human laryngeal squamous carcinoma in nude mice by hypoxia inducible factor-1α (HIF-1α) anti-sense oligodeoxynucleotide. [*Chinese Archives of Otolaryngology-Head and Neck Surgery*](http://202.201.7.11/kns55/loginid.aspx?uid=aDNKRDFBMmdLKzcwRkZiVHNzT3crL3NjajF0MU1EZHBHeTdHM2cwdTA0UHhTZ0p2&p=Navi/Bridge.aspx?LinkType=BaseLink&DBCode=cjfq&TableName=CJFQbaseinfo&Field=BaseID&Value=EBYT)27（5）:586-589
107. Li Z, Liu LC, Wen DS, Liang X, Guo H, Zhang M, Cheng XL (2012)Adenovirus mediate antisense microRNA-21 inhibiting tumor growing of bladder cancer on nude mouse.*Chinese Journal of gerontology*27（5）:1158-1159
108. Li Z, Wei W, Liu LC, Guo H, Zhang M, Yang X, Huang HL (2010)The cure effect of rAV-Tumstatin viral vector on bladder tansplanted tumor in nude mice.Chinese *Journal of immunology*17（11）:438-445
109. Li ZY, Gao MH, Wang JH, Qu T, Cheng LM, Wang ZM, Zhang QW (2011)Inhibitory effect of total bufadienolides from toad venom against H22 tumor in mice and their metabolites.*China Journal of Chinese Materia Medica*l4（31）:2987-2992
110. Lian C, Wang XT,Xie YB, Xiao Q(2011)Effects of Cdx2 Overexpression on Growth and Metastasis of Transplanted Tumor of Human Gastric Cancer in Nude Mice.*Cancer Research on Prevention and Treatment*36（21）:400-402
111. Liang W, Bao HY (2010)Antitumor active constituent in fruiting body of Phelfinus yamanoi against Hepatoma H22 cell.*Institute of Mic robiology*39（4）:630-635
112. Liang Y, Liu CL, Zhang J, Yang XL, Zhu J, Shen Y, Gu XP, Ma ZL (2011)Participation Of Spinal CaMKII-NR2B signal pathway in the Development Of bone cancer Pain in mice.*Chinese Journal of Behavioral Medicine and Brain Science*30（4）:315-317
113. Liang ZX, Qiang YG, Liao YH (2012)Feasibility of cartilage link protein of hyaluronic acid for defining radiotherapeutic target volume in a mouse model of lung tumor.*Journal of Southern Medical University*21（4）:301-305
114. Liao SJ, Hu XJ, Jang XF, Han LF, Xia X, Wang W, Wang CY, Lu YP, Wang SX, Ma D (2012)Effect of HPV16 peptide vaccine in combination with paclitaxel-cisplatin chemotherapy on cervical cancer in vitro and in vivo.*National Medical Journal* 32（3）:3037-3038
115. Liao YL, Sun JC, Chang SF, Yan Y, Guo J, Li M, Wang ZG (2010)LHRHa-targeted microbubbles with ulrasound irradiation enhance the inhibition of cisplatin on xenografted ovarian carcinoma.*Chinese J ultrasound Med* 90（43）:680-683
116. Lin LZ, Wang SM, Zhou JX,(2011)Effects of Yiqi Chutan Recipe on Tumor Growth,Survival Time and Expressions of PRDX-1 and PRDX-6 in Lewis Lung Carcinoma Model Mice with Pi-deficiency Syndrome.[*Chinese Journal of Integrated Traditional and Western Medicine*](http://202.201.7.11/kns55/loginid.aspx?uid=alNRbE1DMmU0ZGJPai9QMjdVZXlxOWJSOU1uenc2S2ZLTW15eWJoZ2xmT1hITjBL&p=Navi/Bridge.aspx?LinkType=BaseLink&DBCode=cjfq&TableName=CJFQbaseinfo&Field=BaseID&Value=ZZXJ)27（8）:99-102
117. Liu AJ, Ma SJ, Zhen GQ, Gao Y, Zhang QY, Li YC (2011)Immune protective effects of breast cancer vaccine on tumor-bearing mice.[*Immunological Journal*](http://202.201.7.11/kns55/loginid.aspx?uid=alNRbE1DMmU0ZGJPai9QMjdVZXlxOWJSOU1uenc2S2ZLTW15eWJoZ2xmT1hITjBL&p=Navi/Bridge.aspx?LinkType=BaseLink&DBCode=cjfq&TableName=CJFQbaseinfo&Field=BaseID&Value=MYXZ)*.*31(1):938-940
118. Liu AJ, Zhang QY, Zheng GQ, Dong H, Fang QQ(2011)Teng AG,Wang WH(2011)Immune-activation Effects of Carotene Degradants on H22 Tumor Cells.[*Modern Food Science and Technology*](http://202.201.7.11/kns55/loginid.aspx?uid=alNRbE1DMmU0ZGJPai9QMjdVZXlxOWJSOU1uenc2S2ZLTW15eWJoZ2xmT1hITjBL&p=Navi/Bridge.aspx?LinkType=BaseLink&DBCode=cjfq&TableName=CJFQbaseinfo&Field=BaseID&Value=GZSP)26（11）:483-485
119. Liu S, Xin Y, Zhang XH, Wang ZY, Luo L, Ge CY, Zhang L, liu Jj, Cao RY (2012)H22 tumor cell lysate plus adjuvants can effectively induce anti-tumor immunity.*Journal of biology*28（5）:34-38
120. Liu CL, Ma ZL, Liang Y, Peng LY, Ren BX, Liu XJ, Gu XP (2010)Effect of intrathecal injection of KN93, a potent inhibitor of CaMKⅡ, on pain behavior in a mouse model of bone cancer pain.*Chinese Journal of behavioral medicine and brain sciencn*19（10）:867-869
121. Liu CH, Xiao C, Zhi XP, Xu YH (2010) TUNEL determination of apoptosis eff ects induced by caf feic acid Ge on rat cervical carcinoma U14. Lab Med Clin 7(20):21177-21178
122. Liu GR, Jia SQ, Yan MR, Su XL (2010) Influence of anti cancer bioactive peptide on P53 in experimental gastric cancer. Acta Acad M ed NeiM ongol 32(2):208-210
123. Liu H, Han YL, Ding H, Li ZW (2010) The Antitumour E ffect of Eupolyphaga Fibrinolyric Protein on S180 and H22 in vivo. Lishizhen Medicine and Materia Medica Research 21(9):2140-2142
124. Liu H, Yang F, Yu ML, Wang X, Cheng X, Jiang ZW (2011) Decreasing toxicity and synergistic effects of total glucosides of paeony on tumor-bearing mice treated by cyclophosphamide. J Bengbu Med Coll 36(9):917-920
125. Liu HT, Yang TM, Qian ZY, Guo K, Zhang LG (2011) In vivo and real- timemon itoring research of rats w ith glioma in radiofreqency ablation by near- infrared spectroscopy technology. JSoutheastUn iv ( M ed SciEd i ) 30(3):436-440
126. Liu H, Lin Y, Jiang JH, Wei JN, Lin QM, Lin YP, Zhou RX (2010) Effect of melatonin on CD4+CD25十regulatory T cells expression in thymus and spleen of mouse with gastric cancer. Journal of anatomy 33(6):725-728
127. Liu L, Yang JP, Zhang ZW (2011) The role of P38 mitogen-activated protein kinase in rats with bone cancer pain. Chinese Journal of Pain Medicine 17(7):432-435
128. Liu LB, Ma T, Chen FY, Xie H, Xue YX (2011) Effect of bradykinin on activity and expression of NF-κB in microvessels of brain glioma of rat. Progress of Anatomical Sciences 17(4):389-392
129. Liu LB, Xue YY, Wang P (2010) Effect of Bradykinin on the mRNA expression of occludin and ZO-1 in rats with brain glioma. Journal of China Medical University 36(7):497-500
130. Liu LM, Liu HG (2010) Ant-i hepatoma activity of nitidine chloride and its effect on topoisomerase. Chinese Pharmacological Bulletin 26(4):497-500
131. Liu L, Xu HX, Wang WQ, Tang ZY (2012) Inhibition of epithelial mesenchymal transition represses metastaslic potential of hepatocellular carcinoma enhanced by hepatic arterial occlusion in mice. Chin J Gen Surg 27(2):123-126
132. Liu LL, Chang LM, Zhang Q, Zhang WJ, Li XY, Wang TJ (2010) Effect of IFN-gamma-endostatin gene therapy in combination with X-rays on inhibition of primary breast tumor growth and lung metastases in a murine model. Chin J Redial Med Prot 30(4):387-390
133. Liu NN, Zhou LL, Yin PH, Wang Y, Fan ZZ, Li Q (2011) Effect of Jianpijiedu recipe on microvessel density and Cyc looxygenase-2 Expression in Helicobacter pylori induced gastric cancer. C JITWM 31(5):647-652
134. Liu S, Wu CY, Chen XF, Yang SF, Sun XY (2011) Inhibitory acting mechanism of Psoralen-Osthole on bone metastasis of breast cancer-an expatiation viewing from OPG/ RANKL / RANK system. C JITWM 31(5):684-689
135. Liu SL, Yang JP, Wang LN, Liu L, Li CF, Ren CG, Zhou J, Li W, Jiang S, Ma ZN, Qiu QC (2010) Changes in expression of Toll-like receptor 4 and its downstream cytokines in spinal cord in a rat model of bone cancer pain. Chin J Anesthesiol 30(2):165-169
136. Liu SF, Wang TT, Gan Y, He HM, Li Q, Li WL (2012) Effects of phycocyanin on immune function in Hepa1-6 cell and tumor-bearing mice. Chin J Public Health 28(3):342-343
137. Liu W. The inhibition and detecting of mechanism of transplanted carcinoma cell growth by N3-o-toluyl-fluorouracil on.Chinese Journal of Pharmaceutical. 2011, (12)
138. Liu W, Zhao ZZ, Zhao LH, Jin XQ (2011) Transplantation of hMDR1-transferred bone marrow mononuclear cells and chemotherapy in breast cancer xenograft in mice. J Third Mil Med Univ 33(15):1551-1554
139. Liu JX, Cao F, Pu HL, Gao F, Yang H, Tian YK (2011) Chang in 5-hydrotryptamine level in spinal dorsal horn in a rat model of tibial bone cancer pain. Chin J Anesthesiol 31(6):695-698
140. Liu X, Li G, Li Q, Tian WD, Zhang W, Chen HH, Li XP (2011) Enhancement of target gene expression by recombinant adeno-associated virus combined with recombinant adenovirus in vivo. J South Med Univ 31(1):44-47
141. Liu W, Ma YM, Sun T, Ni CS, Liu YR, Sun BC (2010) Study on IFN-γ/TGF-βLevel in a model of acute inflammation and melanoma. Chinese clinical tumor 37(2):61-64
142. Liu Y, Hou HX, Liu DR, Chen DH, Qing CM, Li W (2010) Enhancement Effect of emodinon radiosensitivity of human nasopharyngeal carcinoma transplanted in nudemice. Chin Phim J 4517):1331-1334
143. Liu ZJ, Shen Z, Zhao H, Du XF, Feng T, Li HT, Lin LJM, Yao Y (2011) Effect of adenovirus-associated virus carrying kringle 1 domain of hepatocyte growth factor gene on the growth of human prostatic carcinoma xenograft in nude mouse bone. Tumor 31(7):585-590
144. Liu Y, Chen H, Bi XY, Liu ZC, Yuan CJ (2010) The morphology study in apoptosis effect of methypmercuric chloride on brain neuroglioma. Chinese Journal of Gerontology 30(5):609-610
145. Lu L, Chen K, Liu B, Wang ZY, Ge CY, Hou J, Jin L,Xing Y, Cao YY, Liu QJ (2012) Anti-Tumor effects of B16F10 tumor cell lysate vaccine in mouse melanomal. Pharmaceutical Biotechnology 19(3):232-237
146. Luo CL, Zhong L, Gu YZ, Zhou RY, Zhao X (2012) Experimental study on anti-tumor effect of sijunzi decoction in mice with transplanted primary liver cancer. Lishizhen Medicine and Materia Medica Research 22(12):2857-2859
147. Lv J, Wang GH, Deng Y, Cao XN, Lai SY, Tao DD, Hu JB, Gong JP.Inhibitory effects of the cell-permeable TAT-N24 fusion peptide on the growth of S180 ascites tumor. Act a Med Univscitechnol Huaz hong 39(3):369-371,380
148. Ma C, Kuang AR, Huang R, Tang GS (2011) Anionic long circulation liposomes mediated antisense scintigraphy in tumor-bearing rats. Journal of Biomedical Engineering 28(2):233-237
149. Ma K, Ma ZG, Long YM, Zhou LP, Chu GQ, Bian J, Zhou H (2012) Effects of hui medicine aikangfang on metastasis and bcl-2 expression of lung cancer in C57 mice. Cancer Prevention and Control Research 39(7):769-772
150. Ma K, Ma ZG, Zhang LN, Zhou LP, Bian J, Zhou H (2012) Effects of Hui medicine aikang fang for lewis lung carcinoma inhibition and mutant p53 expression. CJTCMP 27(8):2158-2160
151. Ma WH, Wang J, Yang WD, Li GY, Ma XW, Wang Z (2010) Biodistribution and SPECT imaging of 99Tcm labeling NGR peptide in nude mice bearing human HePG2H hepatoma. Journal of Isotopes 25(1):27-32
152. Ma ZG, Ma K, Zhou LP, Bian J, Zhou H (2012) Effective of Hui Medicine aikangFang synergism chemotherapy to Lewis Lung carcinoma cell ultrastructure.Journal of Ningxia Medical University 34(3):201-203
153. Meng XT, Lin C, Mei J, Wang HJ, Ma F, Zhang JL, Zhang Y, Qian HL (2011) In vivo imaging system in detecting expression and distribution of replicationdeficient adenovirus carrying luciferase in mice. Chin J Cancer Biother 18(4):389-393
154. Mi YX, Li YC, Long YH (2010) Antitumor effects of radioiodine labeled KH901

on nude mice bearing hepatoma. Journal of Biomedical Engineering 27(2):389-394

1. Nong XL, Li H, Xia Y (2011) In vivo short hairpin RNA interference of vascular endothelial growth factor on drug-resistant tongue cancer cells. Chin J Stomatol 46(1):15-19
2. Ou YWW, Gao FP, Wang LF, Xie XX, Zhou J, Zhang YH, Cai YY, Liu JY, Chen HH, Pan L, Liu YP, Li JT (2010) Thermoseed induction heating on mammary orthotopic transplantation tumor in rats. Technology Review 28(17):26-30
3. Pan CW, Tang XY, Wu TT, Sun J, Can H, Hu MM, Wang HF, Shao Y, Tu FZ, Shen ZJ (2011) In vivo bladder cancer growth inhibition by a phosphatidylinositol 3-kinase inhibitor ( LY294002). J Cont em p U rol Reprod Oncol 3(3):159-162
4. Pei J, Wei H, Liu ZD, Yu YM, Ni CR, Wu HG (2010) Effects of moxibustion on the expression of IL-1, IL-2, IL-6 mRNA and protein in the cerebral cortex in tumor bearing mice. Acupuncture Research 35(4):243-249
5. Pei XK, Cai M, Zhou HM, Liu C, Xu LJ, Huang JM, Chen ZH, Shi PY (2010) Regulatory ef fect of neuropilin-1 positive T cells in the tumor immunity. Chin J Cancer Prev Treat 17(9):641-643?
6. Pei J, Tang Q, Pan YF, Chen W, Huang RF, Zhang YD (2011) The effect of Fe304 nanometer magnetic fluidinduced hyperthermia on implanted lver cancer in nude mice. Chin J Gen Surg 26(6):237-240
7. Peng LH, Zhou J, Huo F, Zhan SY, Zhang Q, Su CQ, Qian QJ (2011) Treatment of hepatocellular carcinoma with gene -viral therapeutic system CNHK300-mIFN-γ. Guangdong Medical Journal 32(22):2913-2916
8. Peng Y,Song XY, Shen MH (2012) Inhibitory Effect of hericium erinaceus polysaccharide on hepatoma-22 (H22) tumor bearing mice. Food Science 33(9):244-246
9. Ren BX, Gu XP, Liu ZL, Wang D, Ma ZL (2011) Expression changes of metabotropic glutamate receptor 3 and 5 in spinal code in a mouse model of bone cancer pain. Chinese Journal of Pain Medicine 17(6):361-365
10. Ren BX, Gu XP, Zhu W, Zheng YG, Liu ZL, Wang D, Ma ZL (2011) Intrathecal administration of metabotropic glutamate receptor subtype 5 antagonist on pain behavior and spinal astrocytes activation mouse mod of bone cancer pain. Chin J Behav Med Brain Sci 20(4):295-297
11. Ren BX, Ma ZL, Jin YQ, Zhu W, Xie WB (2011) Effect of Shentong Zhuyu decoction on pain behavior and spinalcord astrocytes activation in a mouse model of Osteocarcinoma Pain. C JITWM 31(3):381-385
12. Ren CG, Wang LN, Li W, Zhou J, Yang JP (2010) Effects of intrathecal injection of TNP-ATP on mechanical hyperalgesia in rats suffering from bone cancer pain. Medical Journal of National Defending Forces in Southwest China 20(6):581-583
13. Shen KP, Wang HY, Hu B, Liu W, Hu SY (2010) Antitumor effect of weichangan decoction on subcutaneously implanted human gastric cancer in nude mice. Journal of LiangningTraditional Chinese Medicine 39(2):215-217
14. Shen YL, Xu YH, Gu XY (2011) Effect of Wnt5a-Modified bMSCs on hematopoiesis and on the growth of leukemia cells in geukemia mice. Chinese Journal of Clinical Oncology 38(4):811-816
15. Shen YH, Chen M, Shen WD, Huang SL, Zhang LL, Zou XP (2011) Effect of proton pump inhibitors on cancer cachexia in nude mice bearing SGC-7901 gastric adenocarcinoma1. Chin J Gastroenterol 6(1):11-14
16. Sheng SH, Yu HQ, Liu TJ, Liu S, Zheng YH, Hu XL, Song XF (2011) Anti-tumor activity of biodegradable polymer-paclitaxel conjugate micelles on H22 liver cancer mice models. Chinese Journal of Applied Chemistry 28(11):1280-1285
17. Sheng SH, Yu HQ, Liu TJ, Zheng YH, Jing XB (2011) Targeting effect of amphiphilic copolymer micelles observed by fluorescent imaging in H22 liver cancer-bearing mice. Journa l of Jilin University ( Medicine Edition) 37(3):418-421
18. Shi J, Chen Y, Chen WH, Lin DH (2010) Tumor inhibiting effect of etoposide long􀀁circulating liposomes. Chinese Journal of New Drugs 19(20):1881-1884
19. Shi HY, Tian Y, Luo S, Wang SJ, Zhu FP, Jin LX, Wang JD, Lu GM (2012) Monitoring tumor response to antlangtogenic treatment by Integrating of dynamic contrast enhanced MRI，diffusion weighted imaging and optical imaging in animal model. China J of Radiol 42(3):269-274
20. Si J, Zhang JY, Tang HW, Li JY (2010) Inhibitory effect of Chuanhong Baliu Paste on mouse S180 tumor and its apoptotic induction. CJTCMP 25(7):1015-1018
21. Si TG, Gou Z, Wang HT, Liu CF, Yang M (2011) Experimental study on the immune funtions of splenic dendritic cells after a combined therapy of cryoablation and granulocyte macmphage-cology stimulating factor for prostate cancer. Natl Med J China 91(17):1184-1187
22. Song DY, Yue W, Wei D, Lin SK, Sun C, Deng JH (2010) The apoptosis and anti-vascular effects of sonodynamic chem istry therapy on glioma cells. JApoplexy and Nervous Diseases 27(12):1082-1085
23. Song DY, Yin LC, Lin SK, Wang L, Li JH (2011) The apoptosis and related gene expre ssion of sonodynam ic chemistry therapy on glioma cells. Chinese Journal of Gerontology 31(3):426-429
24. Song DY, Yue W, Yin LC, Wei D, Lin SK, Li C, Li JH (2011) Killing effect of sonodynamic therapy on rat glioma cells. Chin JMinim Invasive Neurosurg 16(8):358-361
25. Song Y, Li M (2011) Inhibition effect of IP6 on subcutaneous transplantation of colon cancer in nude mice. Chin J Cancer Prev Treat 18(23):1821-1824
26. Song Y, Shen J, Xu F (2010) Synergism of antitumor effects Oil ovarian carcinom using autocatalytic caspase-3 combined with flavopiridol. Chin J Obstet Gynecol 45(10):781-786
27. Song YP,Liu Z,Zhong Y,Kang CS,Xu P,Han L,Zhang AL,Wang GX,Jia ZF,PuPY (2010) The Effects of combined therapy of Phosphatidy linositol 3p-K inase inhibitor and Ad-PTEN in human gliom a nude mice mode. Chin J Nerv Ment Dis 36 (002):104-107
28. Su N,Dong Q,Lu HT,Yang ZM,Jiang Z,Hao XW,Sui AH (2012) Inhibition of the growth of neuroblastoma by CXCR4-siRNA. Chin J Pediatr Surg，January 33(001):54-5
29. Sun D,Cai JM,Zhu BR,Cheng Y,Li BL,Cui JG (2011) Inhibitory effect of STAT3 antisense oligodeoxynucleotide against human adenocarcinoma of lung derived from A549 cell in nude mice. Academic Journal of Second Military Medical University 32(1):76-79
30. Sun EL,Fan XD,Wang YY,Han RF (2011) Immune response of recombinant IFN-a-2b-BCG treatment in mice bladder cancer.Chin j Urol,January 32(001):38-41
31. Sun J,Liu L,Wu QH,Nie Q,G HL,Yang ZX,H Y (2010) Biodistribution and anti2cancer potential of 131 I2172AAG in NSCLC xenograft2bearing nude mice. Chin j cancer prev treat 17 (03):177-181
32. Sun T,Yang W,Cao JP,Liu FJ (2011) Anti- tum or effect of RNA interference Silencing Survivin Gene combined with X - ray irradiation on human hepatoma xenograft in nude m ice. Chin J Rad iol Health 19(4):398-400
33. Sun WJ,Yu HJ,Neng J,Xu Y,Liao ZK,Zhou FX,Xie CH,Zhou YF (2011) Anti-tumor effect of adenovirus-mediated suicide gene therapy under control of tumor-specific and radio-inducible chimeric promoter in combination with Y-ray irradiation in vivo.Chin j Radiol Med Prot 31(1)：6-9
34. Sun XJ,Zhang YY,Jia Q,Wang ZM,Wang ZX,Zhang WD (2011) Effect of polypeptide extract from scorpion venom (PESV) with chemotherapy inhibited angiogenesis of lewis lung carcinomas.China journal of Chinese meteria medica 36 (12):1644-1649
35. Sun YE,Ma ZL,Zheng YG,Zhang J,Yang XL,Zhu J,Gu XP (2012) Effect of intrathecal injection of magnesium sulfate on pain behavior in bone cancer pain mouse.Chin J Behav Med and Brain Sci 21(4):306-310
36. Sun YL,Xu C,Su CQ,Ma JX,Gao J,Man XH,Li ZS (2012) Recombinant adenovirus-mediated Hsp70 gene expression inhibits tumor growth in a rat xenograft model of pancreatic cancer. World Chinese Journal of Digestology 20(1):15-21
37. Suo YY,Yang WD,Ma XW,Wang J (2011) Labeling of NGR Peptide With 188 Re and Its Biodistribution and SPECT Imaging in Tumor -bearing Nude Mice. Journal of Isotopes 24 (02):77-82
38. Tan G,Xu L,Fang YQ,Tao G,Wang ZY,Tan Y (2010) Anti- tumor Effect of Specific Cytotoxic T Lymphocytes A ctiviated by Dendritic Cells Pulsed with K - ras Antigen on Pancreatic Cancer. Chinese Journal of Clinical Oncology 37(8):421-425
39. Tian T,Zhang PT,Liu YH,Yu MW (2010) Effects of lignum sappan on growth and metastases of lewis lung carcinoma at different phases in C57BL/6 mice. Chinese Journal of Integrated Traditional and Western Medicine 30(07):733-737
40. Tong YX,Wang GH,Sun L,DengY,Cao XN,Lai SY,Li XL,Hu JB,Gong JP (2010) The fusion
41. Peptide inhibits the growth of nude mice colon xenograft tumor. Chinese Journal of Experimental Surgery 29(002):233-236
42. Tu YY, Fu JF,Wang BL,Cao YZ (2010) The study of polypeptide from agkistrodon acutus venom inhibitory effects on U251 glioma subcutaneous model in nude mice.SHAI XI Medical Journal 39(03):263-273
43. Tuo Y, Du RT, Zhang HP (2010) Effects and mechanism of probiotics L b. casei Zhang for tumor-bearing mice on antitumor. Cancer Research On Prevention and Treatment 37(04):463-465.
44. Wang FB,Chen ZF,Chen DP,Liu K,Yang F,Feng MF,Xie W,Zhu YQ,Xia B (2012) Antitumor immune effect of interleuidn-7 gene for mouse mammary cancer treated with direct in-tratumoral injection. Chinese Journal of Experimental Surgery 29(001):64-66
45. Wang FB,Chen ZF,Chen DP ,Yang F, Liu K ,Feng MF,Xie W,Zhu YQ,Xia B (2012) Enhancement of antitumor effect On breast cancer by interleukln-7-induced interferon-ɤ production in CD8+ T cells. Chinese Journal of Experimental Surgery 29(002):233-236
46. Wang Q,Jia N,Yang DX,Wang SS,Guo WJ,Yuan SJ (2012) Evaluation of inhibition effects of T peptide on postsurgical residual tumor growth. military medical science 36 (05):357-361
47. Wang B,Yuan T,Zhang YW,Wang ZY,Qing YL,Xue XZ,Chang YQ (2012) The antitumor effect of intestinal polysaccharides in sea cucumber Apostichopus japonicus. Journal of dalian ocean university 27 (03):195-199
48. Wang CG,Liu BL,Ceng L,Liu YL,Liu JX,Liu XT,Wang W (2011) Changes of moleculars involved in PI3K/AKT pathway in emodin-induced apoptosis of human leukemia K562 cells in nude mice.Tumor 31(7):573-579
49. Wang CG,Liu BZ,Jin DT,Wang C,Wu Y,Zhu D,Zhong L (2010) Inhibitory effect of emodin on the subcutaneously transplantedtumor of humanK562 cell innude mice. Chinese Journal of Hospital Pharmacy 30 (03):179-182
50. Wang CG,Liu BZ,Jin DT,Wang C, Zhu D,Wu Y, Zhong L (2010) Inhibition of emodin on xenografted human K562 cells in nude mice and regulation on relationship of Caspase-3 and Caspase -9 expression. Chinese Traditional and Herbal Drugs 41(05):51-756
51. Wang F,Pan SY,Xu T,Huang PJ,Xu J,Xia WY,Lu YC,Peng Y,Qin XY,Geng Y,Sun RH,Huang L (2011) Experimental study on the inhibitory effect of monoclonal antibody against human nonsmall cell lung cancer on lung adenocarcinoma. Acta universitatis medicinalis Nanjing (Natural Science) 31 (7): 940-944
52. Wang G，Huang Y,Feng KK,Li YH,Wang Zl (2011) Effect of AAV-AS and cytoxan on subcutaneous glio ma in rats. Shandong Medical Journal 51（2):10-12
53. Wang G,Zhou J,Feng KK,Tian L (2011) Anti-angiogenesis effect of adeno-associated virus-mediated recombinant angiostatin combined with celastrol on intracranial C6 glioma in rats.Tumor 31 (10): 875-880
54. Wang HN,Shen KP, Hu B,Liu W (2011) Influence of Weichangan Decoction on the Expressions of p- AKT and Twist Proteins in Xenograft Nude Mice Model of Human Gastric Carcinoma SGC- 7901. China journal of Chinese medicine 1 (12):1409-1411
55. Wang H. (2010) A Empirical Study on Prediction of Colorectal Cancer Metastatic Potential Chinese Journal of Nuclear Medicine 7(2).
56. Wang HM, Ge W, Cao DD, Zheng YF, Jie FF, He D, Hu K, Zhang JZ (2010) Effects of the expression of VEGF and tumor Growth in mice of Lewis lung cancer by rh-Endostatin (YH-16) in combination with radiotherapy. Chinese Journal of Microcirculation 2010,20(4):19-21.
57. Wang LF,Shan BE,Shan TQ (2010) Effects of trite rpenes compound of cortex periplocae on regulatory T cells function in N - nitrosomethylbenzylamine – induced esophageal tumorige nesis in F344 rats. Canceration, distortion, mutation Publications 22 (3) :206-209
58. Wang LF,Liu LH,Ma LM,Meng FR,Shan TQ,Shan BE (2010) Triterpenes compound extracted from cortex periplocae inhibits tumorigenesis of esophageal cancer Eca109 cells in nudem ice and relatedmechanisms. Chinese Journal of Cancer Biotherapy 17 ( 6):620-624
59. Wang LF,Lu A,Meng RF,Cao Q,Ji X,Shan BE (2012) Inhibitory effects of triterpenes compound of cortex periplocae on N-nitrosomethyl-benzylamine-introduced rat esophageal tumorigenesis. Cancer Research On Prevention and Treatment 39 (1):23-27
60. Wang LN,Yang JP,Ji FH,Wang XY,Zuo JL,Xu QN,Jia XM,Zhou J,Ren CG,Li W (2011) The role of brain-derived neurotrophic factor in pain facititation and spinal mechanism in rat modle of bone cancer pain.National Medical Journal of China 91(17):1188-1192
61. Wang L,Chen Y,Zhu SJ,Shi HT,Wang P,Liu QH (2012) Metabolism and distribution of chlorin e6 in S180 tumor-bearing mice.Journal of Shaanxi Normal University 40(2):71-75
62. Wang M,You Y,Yang TM,Qian ZY,Bao MF (2012) Research on the application of near-infrared spectorscopy technology in laser insterstitial thermotherapy for rats with glioma.Journal of Southeast University 31(2):131-135
63. Wang SM,Lin LZ,Xiong SQ,Zhou JX,Sun LL (2011) Effect of Yiqi Chutan Recipe on the Expression of Matrix Metalloproteinases in Lung Cancer.Traditional Chinese Drug Research & Clinical Pharmacology 22(2):135-138
64. Wang SS,Zhai XF,Li B (2011) Effect of Cinobufacini Injection on the Tumor Growth of Tumor Bearing Rats of Different Constitutions.Chinese Journal of Integrated Traditional and Western Medicine 31(8):1101-1103
65. Wang WJ,Zhang NM,Du Y,Li H,Zhang D,Yang YX (2011) Inhibitory effect of celecoxib on colorectal adenomas in rats.Chinese Journal of Experimental Surgery 28(1):39-41
66. Wang XW,Tao ZH,He HH,Zhou JM,Tang ZY,Wang L (2010) Interferon-d significantly inhibits the hepatocyte growth factor and vascular endothelial growth factor expression in the hepatocellular carcinoma of nude mice model with highly metastatic potential.Chinese Journal of Experimental Surgery 27(12):1812-1814
67. Wang XJ,Zhang ZZ,Shao YJ,Quan SX,Li HX (2010) The Effect of Tumor—targeted Gene Delivery of NGR／LPD Complexes OH Telomerase in the MCF-7 Cells in vivo．Journal of Medical Research 39(8):30-34
68. Wang XT,Li L,Qian Q,Xie YB,Xiao Q (2010) The effects of E2F transcription factor 1 on human gastric subcutaneous tunlor of nude mice.Chinese Journal of Experimental Surgery 27(2):207-209
69. Wang YZ,Zeng JM,Wei R,Li CL,Xiao Q,Chen XM,Feng WL (2010) Effect of decoy RNA of poly(rC)-binding protein E2 on the tumorigenic ability of 32D-BCR/ABL cells in mice.Tumor 30(7):561-565
70. Wang YM,Wang LJ,Yang RJ,Cai JY,Wu LH,He XX (2011) The effect of siRNA targeting MIF on the growth of colorectal cancer xenografts and the life quality of tumor-bearing mice.Chinese Journal of General Surgery 26(5):376-380
71. Wang Y,Tian YJ,Wan H,Li JJ,Li DZ,Ma JY,Wu WH,Yin LX,Jiang J,Wan WQ,Zhang LW (2012) Comparison of differences in invasiveness of C6 brainstem glioma between juvenile and adult rats.Chinese Journal of Minimally Invasive Neurosurgery 17(2):82-85
72. Wang YY,Li DG,Wu HY,Liu Q,Zhang H,Du LQ,Lu L,Meng AM,Wang RQ,Zhang LA (2010) Effects of E838 Combined with Cyclophosphamide on Leukemia L1210 Cell.Cancer Resaerch on Prevention and Treatment 37(2):129-131
73. Wang YY,Li DG,Wu HY,Zhang H,Du LQ,Lu L,Meng AM (2010) Inhibitive efects of E838 com bined radiotherapy and chem otherapy on m alignant tumor in IRM -2 mice.Chinese Journal of Biochemical Pharmaceutic 31(3):154-157
74. Wang Z,Fu YJ,Chang H,Zhao CJ,Mi MT (2010) Inhibitory effect of anthocyanin extract of mulberry fruit on growth of breast cancer MDA-MB-453 cells.Acta Academiae Medicinae Militaris Teriae 37(2):988-990
75. Wang CS,Li H,Gao CF,Chen YP (2010) iASPP on apoptosis in breast cancer cells which expressed wild type p53.Chinese Journal of Pathophysiology 26(2):282-286
76. Wang ZP,Zhang WD,Wu LC,Jia Q,Wang ZX,Zhang YY,Wang Y (2010) Inhibitive effect of polypeptide extract from scorpion venom on repopulation in H22 tumor cell during chemotherapy.China Journal of Chinese Materia Medica 35(1):108-113
77. Wang ZP,Zhang WD,Wu LC,Jia Q,Wang ZX,Zhang YY,Zhang JP (2011) Inhibition of polypeptide extract from scorpion venom on repopulation in Lewis lung adenocarcinoma during chemotherapy.Chinese Traditional and Herbal 42(1):103-107
78. Wang ZM,Liu Y,Chen KM,Gong J,Zheng YF,Zhang LY,Liu FJ (2011) Early therapy monitoring of 125 I seed interstitial implant in a pancreatic cancer xenograft by F-FDG Micro-PET／CT. Chinese Journal of Radiological Medicine Protection 31(3):256-259,289
79. Wei M,Zheng SZ,Ma H,Lv Y (2011) Study of lyceum barbarum polysaccharides on interventing IGF-I，IGFR and IGFBP-1 levels of Human ovarian carcinoma in nude mice.Journal of TCn University of Hunan 31(9):19-22
80. Wen ZH,Su XL (2011) Influence of p53 and Bcl-2 expression of anti-cancer bioactive peptide on experimental gastric cancer.Medical Recapitulate 17(10):1557-1559
81. Wu HJ,Hu YY,Li WH,Chen H,Xiao YP,Xin Y (2011) Antiangiogenic Effect of (-)-Epigallocatechin-3-Gallate on Gastric Cancer.Journal of China Medical University 40(6):494-496
82. Wu K, He BC, Zhou QX (2012) Berberine in colon cancer prevention and control of experimental rats and the effect and its and peroxide multiplication correlation of activated receptor gamma expression.Chinese journal of biological science 24(8):952-956
83. Wu KQ, He TP, Liu Y, Lv YN, Chen G, Lai BS, Liang NC (2011) Half of the flag cut inflammation factor expression of lotus extract 5 f the effect of antitumor activity of mice S180 sarcoma.Journal of guangdong medical college 29(9):355-358
84. Wu KF, Lv YN, Liu Y, Li L, George, G． Chen, Liang NC (2011) Half of the flag extract 5 f - Na salt injection preparation and its inhibition effect on type S180 sarcoma in mice.When Jane GuoYi characters 22(3):546-549
85. Wu Q, Yu J, Jin NZ, Zhao RZ (2011) Soybean isoflavones affect a tumor-burdened go nude mouse ovarian tumor .GrowthJournal of nutrition 33（5）：510－513,517
86. Wu Q, Wang Y, Zhou LH, Liu NN, Sun J, Fan ZZ, Li Q (2011) Induction of nude mice spleen and detoxification party of human gastric cancer cells apoptosis related gene expression research.Journal of Chinese medicine 26(4):701-705
87. Wu ZC,DongJ, Xiang CW, Zhou GH, Huang Q, Lan Q, Wu XY (2010) For the mo thiazole amine slow-release microspheres experimental research for the treatment of glioma subcutaneous transplantation tumor. Journal of suzhou university 30( 5):910-914
88. Wu ZY, Du XH, Xu YX, Li L, Liu JC, Wang JJ, Li ZX (2010) Cytotoxic T lymphocytes and cytokine induced killer cells in Dutch people the migration of gastric cancer in nude mice and distribution.Chinese medical journal 90(6):403-406
89. Xi SY, Zhang Q, Liu CY, Xie H, Yue LF, Li WD, Zang BX, Gao XM (2012) Safflower HSYA components of human gastric adenocarcinoma BGC - 823 nude mouse transplantation tumor VEGF protein expression and KDR and hypoxia induced factor of influence.Journal of Chinese medicine 27(1):82-87
90. Xi SY, Zhang Q, Liu CY, Xie H, Yue LF, Zhao YF, Zang BX, Gao XM (2010) Hydroxy safflower yellow pigment A tumor tissue bFGF protein in human gastric cancer transplantation tumor nude mice and the influence of the expression of MMP - 9.China journal of traditional Chinese medicine 35(21):2877-2880
91. Xia JZ, Wu F, Ran LF (2010) Adoptive immunity activation of T lymphocytes after HIFU treatment in a tumor-burdened rat tumor local function change.Chinese medical imaging technology 26(10):1823-1826
92. Xiao Q, Huang C, Fan XH, Song DZ, Liang Y, Gong JL, Wang LF, Liu JL, Lai ZP (2011) New city of 7793 strains inhibit the growth of human colon cancer LoVo cells in nude mice transplantation tumor and its mechanism.Chinese journal of tumor biological treatment 18(2):144-148
93. Xiao XP, Cao F, XU AJ, Gao F, Tian XF, Tian XB, Tian YK (2010) Eff ect of analgesia by intrathecal injection of PKCC-shRNA lentivirus in rat model of bone cancer pain.Journal of clinical anesthesiology 26(5):424-426`
94. Xiao YB, Xie J, Zhang GX, Li J, He YC, Zhang XW, Xu T, Wang XF (2012) Inhibitory effect of CMTM5 on xenografted human prostatic cancer in nude mice.The Chinese male science magazine 18(3):195-199
95. Xu L, Zhang WT, Wang ZP, Jia Q, Zhang YY, Jiang GS (2010) Effect of polypeptide extract from scorpion venom ( PESV) on immune escape of Lewis lung carcinomas.Child journal of chinese materia medica 35(17):2324-2327
96. Xu L, Zhang WD, Wang ZP,Zhang YY, Jia Q (2010) Inhibitive effect of polypeptide extract from scorpion venom( PESV)on the growth and metastasis of Lewis lung carcinomas.Journal Of Shandong University 48(1):82-84
97. Xu WF, Wang F, Chen HM, Lu XL, Yan XJ (2010) Preliminary study on antitumor effects of Fascaplysin and safety in vivo in transplanted S180 tumor of ICR mice .Chinese clinical pharmacology and therapeutics 15( 11):1206-1210
98. Xu WF, Wang F, Chen HM, L XL, Yan XJ (2012) Preliminary study on antitumor effects of Fascaplysin and safety in vivo in transplanted S180 tumor of ICR mice.Chin J Clin Pharmacol T her 41(5):35-39
99. Yin XH, Cheng Y, Cui SF (2010) Bovine spleen peptide for liver cancer tumor growth inhibition in mice.Journal of Inner Mongolia medical college 20(6):832-835
100. Yang B, Cheng Xin, Zhang SZ, Wang B, Han M (2011) Dynamic enhanced MRI evaluation of endothelial inhibition of tumor angiogenesis in colon cancer in mice.Practical radiology journal 27(4):620-623
101. Yang J, Xie YB, Xiao Q, Wang XT, Li L (2011) RNA interference mediated Cdx2 silence on human gastric cancer cell MGC - 803 in nude mice transplantation tumor growth.Chinese journal of modern medicine 21(34):4227-4235
102. Yang J, Li ZH, Zhou JJ, Chen RF, Cheng LZ, Zhou QB, Yang LQ (2010) Preparation and antitumor effects of nanovaccines w ith MAGE-3peptides in transp lanted gastric cancer in mice.Chinese Journal Of Cancer 29( 4): 393- 398
103. Yang LB, Shi ZJ, Guan MQ, Li P, Zhao ZD, Wang J, Xiao J (2010) The functional identification of short peptide binding specifically to osteosarcoma-associated vascular endothelial cells in-vivo.Chinese journal of orthopaedic 35(5): 501-505
104. Yang W, Sun T, Cao JP, Liu FJ, Zhu W, Chen Q (2010) Anti-tumor effect of silencing HIF-1α and survivin genes combined with radiotherapy on human hepatoma xenograft in nude mice.Radiation research and radiation work of art 28(4): 239-243
105. Yang XL, Ma ZL, Zhu J, Cui XL, Liang Y, Shen L, Liu H, Gu XP (2012) Effect of repeated intrathecal injection of ifenprodil on pain behaviors in mice with bone cancer pain.Chinese journal of behavioral and brain sciences 21(3): 228-231
106. Yang YG, Chen XM, Wang XL, Wang C, Hua HY (2012) Experimental Study on Liver Metastases of Colorectal Carcinoma Treated by Recipe of Blood-activating and Stasis－eliminating.When Jane GuoYi characters 23(2): 280-282
107. Yang ZX, Liu L, Wu QH, Zhu XL (2011) Specific binding between IL-11 analogue c(CGIUtAGGSC)and PC-3 cells in vitro.Chinese journal of experimental surgery 28(4): 600-602
108. Yang ZZ, Zhang YS, Li MX, Zeng LL, LI ZP, Wang D, Wang G (2010) Hematoporphyrin derivatives mediated photodynamic therapy of human lung adenocarcinoma cancer cell killing effect of experimental research.Third military medical university journals 2010,32(5): 494-496
109. Yang ZM, Wang DD,LI L, Zhang PX (2011) The Bcl - 2 in SCID mice oleanolic acid induced HL 60 apoptosis regulation function. Journal of jilin university 37(1): 11-14
110. Yao M, Cao HP, Yang JH, Xiao WP, Wang LN, Cheng H, Peng Y.Variation of spinal microglia in a SD rat model of bone cancer pain.The experimental surgical magazine 2010,27(6): 832-834
111. Yao X, Jia LP, Tan HY, Gao FY (2012) Effects of TCM Herbs of Reinforcing Kidney and Strengthening Bones on Bone Destruction in Rats with Bone Metastasis from Breast Cancer.Journal of traditional Chinese medicine 20(4):14-16
112. Yao X, Jia LP, Tan HY, Pan L, Guo YR, Gao FY, Li H, Yan Y (2012) Effects of epimedium brevicornum on cancer pain and osteoclast in rat model of bone cancer pain.Journal of Chinese medicine.Biotechnology drugs 27(5):1266-1269
113. Yao Y, Zhang Y, Zhang F, Hou J, Luo XL, Cao RY, Jin L, Wu J, Liu JJ (2011) Anti Tumor Effects of S-adenosyl- L-Methionine in Mouse Liver Cancer H22 Cells.Biotechnology drugs 18(1):043-047
114. Ye JY, Hu XB, Xing Y, Hou J, Li F, Zhang F, Jin L, Li TM, Liu JJ, Cao RY (2010) Ant-i Tumor Effects of Protein Vaccine against Human Chorionic Gonadotrophin Beta (BhCG).Biotechnology drugs 17(5):386-392
115. Yi H, Du BY, Tan YH, Liu AJ, Luo H, Luo HH, Su JF, Wang HF (2011) Joint Therapeutic Effect of Hepatocarcinoma Suicide Gene Therapy Combined with Liuwei Dihuangwan on Expression of Connexin in vitro.Chinese journal of experimental formulas of Chinese medicine 17(12):114-118
116. Yin WH, Li H, Yang JP, Wang LN, Zheng XY, Xu QN, Wang XY, Zuo YL (2011) Change in T-cell death-associated gene 8 expression in spinal cord in rats with bone cancer pain.Chinese journal of anesthesiology 2011,31(8):935-937
117. Yin LY, Bian XY, Xiao HB (2010) The Study of the Anti － tumor Effect of the Total Saponine of Aralia Elata Seem Leaves in the Rats with H22.The Chinese medicine information 2010,27(3):107-109
118. Yin LY, Bian XY, Xiao HB, Kuang HX (2010) Liaodong Cong MuYe total saponins of H22 tumor-burdened p53 and PCNA protein expression in mice.Journal of traditional Chinese medicine 2010,38(1):18-20
119. Yin XH, Cheng Y, Cui SF (2010) Effect of genistein and daidzein on expression of TNF-a, IL-1a and IL-6 in mouse endometrial cancer.Chinese journal of modern medicine 2010,20(6):832-835
120. Yu L, Liu YZ, Sun SL, Fang F, Gong HW, Chen Q, Ju GZ (2011) Detection on promoter methylation of c-my c gene in thymic lymphomas induced by ionizing radiation.China's public health 2011,27(1):81-82
121. Yu MW, Sun GZ, Qi X, Li DR, Zhang PT, Wu J (2010) Expressions of CD4 + and CD25 + of Tumor-bearing mice and Its Regulatory Molecules Intervened by Caesalpinina Sappan and Caesalpinina Sappan matched Radix Astragali.China's basic medical journal of traditional Chinese medicine 16(5):384-386
122. Yu MW, Sun GZ, Wu J, Li DR, Qi X, Zhang PT (2010) Interventional effects of Huangqi (Radix Astragali) and Sumu ( Lignum Sappan ) and their formulas on expression of spleen regulatory T cells and serum level of cytokines in tumor-bearing mice.Journal of Beijing university of Chinese medicine 33(4):241-245
123. Yu MW, Sun GZ, Zhang PT, Wu J, Wang XM, Yang GW (2011) Different types of invigorate the circulation of medicine and the compatibility of yiqi pills intervention effect of Lewis lung cancer growth in mice.Beijing traditional Chinese medicine 30(11):859-861
124. ABDUZAYIR Abliz, UPUR Halmurat, TURSUN Yusup, KURBAN Abdukadir, BAKRI Iskandar (2011) Effect of Abnormal Savda Munziq on Morphological Structure of Hepatic Tissue in Hepatocirrhosis Phase of Hepatocarcinoma Carrying Abnormal Savda Model.Tech review 29(28):68-73301.
125. Yuan Jh, jiang h, yang xy, li yq (2010) Effects of epigallocatechin gallate OD growth and metastases of colonic cancer.The digest magazine 30(1):18-23
126. Yuan JH, Li YQ, Yang XY (2010) The role ofN rf2 in the expression ofUGT1A and its iso forms induced by EGCG in colon tumor.Basic medicine and clinical 30(12):1257-1262
127. Yun CX, Deng JG, Wang K, Lan TJ, Luo XF, Xie MQ (2010) A tumor-burdened mangiferin on chemotherapy cytokines in mice and T the influence of the level of intracellular second messenger.Journal of guangxi medical university 27(6):829-832
128. Zhan Q, Hu SS (2011) Inhibiting Effect of Anti-VCAM-1 Monoclonal Antibody with Photodynamic Therapy on in vivo Growth of C6 Glioma Xenografts.Chinese journal of medical laser 20(4):204-208
129. Zhang BP, Zhang H, Zhang XW, Zhang GX, Wang XH, Liu Y, Liu B (2011) Effect of Heavy Ion Beam on Concentrations of Trace Elements in Serum of Golden Hamster with Cheek Pouch Carcinoma.*Nuclear physics* 28(2):225-229
130. Zhang DF, Sun BC, Zhao XL, Cui YF, Xu SY, Dong XY, che n (2011) Acceleration of Breast Cancer Growth during Pregnancy in Tientsin Albino 2 Mice.*China of oncology 38(15):871-874*
131. Zhang F, Hu XB, Cao RY, Zhu XJ, Liu JJ (2011) Antitumor effect of trichosanthin onmouse hepatocellular carc inoma H22 cells*.Zhongke medical university* 42( 3): 242- 246
132. Zhang FH, Meng ZW, Tan J (2010) Combination of NF-κB Inhibitor and Docetaxel Enhances Anti-tumor Activity in Anaplastic Thyroid *Cancer Cell Xenograft in Vivo.Tianjin medical* 38(8): 686-688
133. Zhang H, Song YS, Shang C (2011) The immunological therapy effect of combined Ag85A and GM － CSF DNA vaccine in bladder tumor － bearing mice cells.*Modern medicine tumor* 19(6): 1049-1051
134. Zhang HJ, Lu JP, Yang JJ, Zhu J, Li YX, He Q, Sheng J, Zhang SM (2012) By the arterial perfusion of telomerase inhibitors AZT Walker - 256 of rat liver tumor.*Radiology practices* 27(1):17-20
135. Zhang JC, Yang TM (2010) Magnetic nano。iron treatment of rat glioma：an experimental study.*Journal of southeast university* 29(3):254-259
136. Zhang JY, Li HY, Li JY, Si L, Zhang C, Ma CL (2010) Effect of ChineseMedicine Rec ipe on Anti- tumor of Sma ll RatG lioblastoma and theMechanism of Action.*Liaoning journal of traditional Chinese medicine* 37(3):560-562
137. Zhang J,Li H,Wang GS，Jiang N，Yang Y，Chen GH (2010) Effects of L-arginine on tumor growth of human hepatocellular carcinoma in nude mice.*Chinese Journal of Hepatobiliary Surgery* 16(4):283-285
138. Zhang JZ,Hu K,Ge W,Li CH,Zhang YF,Zhang L,Wang HM,Cao DD (2011) Effect of endostar combined with radiotherapy on the expression of aquaporin1 land hypoxia-inducible factor-1 alpha mRNA and growth of mice with lewis lung cancer*.Medical journal of Wuhan University 3*1(1):44-48
139. Zhang J,Yang G,Dan BN,Zhang C,Zhao RN,Liu JH (2010) Antitumor effect of periplocin from Periplocae Cortex on H22 tumor bearing mice and its mechanism.*Chinese Traditional and Herbal Drugs* 41(8):1307-1311
140. Zhang J,Liu LM.Chen LY,Wang P,Wang YQ (2012) Effects on the Pancreatic Orthotopic Transplantation Tumor in Nude Mice by Different Chinese Medicine Therapeutic Methods.*Chinese Journal of Integrated Traditional and Western Medicine* 32(5):657-660?
141. Zhang J,Tang JB,Song MY,Wang B,Li RK,Hou L,Huang YH,Wang M,Song B,Jin YL (2011) Expression of enoyl CoA hydratase 1 reduces cell proliferation and migration in mouse hepatocarcinoma cells. *Chinese Journal of Pathology* 40(12):830-833
142. Zhang SX,Wu HG,Yao ZA,Wang FF,Du LG (2012) Anti-tumor effect and immunoregulatory activity of two marine oligosaccharides on S180 sarcoma in mice.*Current Immunology* 32(1):5-8
143. Zhang FL,Zhang JH,Wang RF,Yan P,Chen LX (2012)Analysis of immune factors on uptake of F-FDG in lymphoma of NOD/SCID mice.*Chinese Journal of medical imaging technology* 28(6):1027-1030
144. Zhang WF,Shuai JH,Li P,Yang R,Li XG,Luo KY (2011) Inhibitory mechanism of iodine一125 particles against the expression of nerve growth factor in breast cancer cells.*Chin J Exp Surg* 28(5):656-65
145. Zhang WF,Wang MC,Luo KY,Li P,Yang R,Zhang ZP,Li XG,Chu Y (2010) Experiment research on 125I interstitial rachytherapy inhabiting the express of bFGF in breast cancer cell.*Chin J Curr Adv Gen Surg* 13(6):426-429
146. Zhang W,Sun HC,Xiong YQ,Zhuang PY,Zhu XD,Tang ZY (2010) Effects of avastin combined with sorafenib in a highly metastatic murine model of human hepatocel1ular carcinoma.*Chin J Exp Surg* 27(2):146-148
147. Zhang W,Sun HC,Xiong YQ,Zhuang PY,Zhu XD,Tang ZY (2011) Enhancement of apoptosis by aspirin combined with sorafenib in a highly metastatic murine model of human hepatocellular carcinoma.*Chin J Exp Surg* 28(10):1620-1622
148. Zhang WJ,Liu Q (2012) Targeting therapy of docetaxel-loaded nanoparticles for mouse hepatic carcinoma xenografts.*Journal of Clinical Medicine in Practic* 16(5):1-6
149. Zhang SM,Sun J,Hu SS (2010) Influence of Photodynamic Therapy Combined with Cisplatin for Brain Glioma on Expression of P-glycoprotein in Blood-tumor Barrier.*Chin J Laser Med* Surg 19(2):69-72
150. Zhang X,Wang JF,Li H,Liu D,Li ZJ,Xue CH (2011) The anti-metastasis effect of chondroitin sulfate isolated from isostichopus badionotus on spontaneous metastasis of mouse lewis lung carcinoma and its mechanism. *Acta Nutrimenta Sinic*a 33(6):597-601
151. Zhang X,Wang JF,Yang YH,Chang YG,Xue CH (2011) Anti-metastasis effect of SC-FUC on spontaneous metastasis of mouse Lewis lung carcinoma and its mechanism.*Chinese Pharmacological Bulletin* 27(8):1098-1103
152. Zhang YX,Wang YL,He YX,Hu YN,Lu XL,Liu EQ (2011) Effects of Saikosaponins-d on STAT3 and COX-2 signal transduction pathway in experimental hepatocarcinoma of rats.*Chin J Gastroenterol Hepatol* 20(8):735-738
153. Zhang YP,Luo J,Ran HT (2012) Detecting on effect of anti-tumor and the side reaction of sonosensitizer-loaded high polymer ultrasound contrast agents.*Chin J Clinicians( Electronic Edition)* 6(9):2399-2402
154. Zhang YP,Ran HT,Wang ZG,Zhang H,Li H,Lang Q (2010) Effect of sonodynamic therapy on H22 tumor-bearing mice using hematoporphyrin-loaded PLGA ultrasound contrast agents. *Chin J Med Imaging Technol* 26(4):593-596
155. Zhang YY,Gao GL,Gao J,Wang F (2011) Expression of sIL-2R,VEGF and CA125 in node mice bearing human ovarian carcinoma induced by adverse psychological stress. *Cancer Research on Prevention and Treatment* 38(4):365-368
156. Zhang Y,Yang XY,Kuang ZS,Xiao C (2010) Inhibitory effect of cinnamon acid germanium on growth of uterocervical carcinoma (U14)cells in mice.*J Chin Exp Pathol* 26(4):467-470
157. Zhang Y,Lu ZY,Wang JJ,Feng WW,Ding JX,Hua KQ (2011) Study on the effects of gonadotropin-releasing hormone analogues in the inhibition of ovarian cancer transplanted tumors and in the protection of ovarian function after chemotherapy on nude mice. *Chinese* *Journal of Obstetrics and Gynecology* 46(12):892-897
158. Zhang Y,Xu JH,Fan ZZ,Sun Y,Zhu YW,Liang F,Han JH,Li CH,Lu H,Sun XW,Guo G (2010) Effect of Jianpi Jiedu Fang on Platinum Resistance Associated Copper-Transporting Protein (ATP7A /ATP7B) in the Orthotopic Transplantation Nude Mouse Mode of Human Colon Carcinoma. *LIAONING JOURNAL OF TRADITIONAL CHINESE MEDICINE* 37(5):942-945
159. Zhang YY,Zhang WD,Wu LC,Wang ZX,Wang ZP,Jia Q,Xu L,Zhang JP (2010) Effect of PESV on dendritic cels maturation in the tumor microenvironment.*JOURNAL OF SHANDONG UN IVERSITY ( HEALTH SC IENCES)* 48(10):34-38
160. Zhao H,Xiang HJ,Cao ZQ (2011) Effect of Kunshen Granule on the Expression of VEGF- C、VEGFR- 3 of Human Gastric Carcinoma in Nude Mice Model.*Chinese Journal of Pharmacovigilance* 8(5):257-260
161. Zhao JM,Sun BC,Liu YR,Zhao XL,Zong WK,Dong XY,Zhao N (2011) The role of caspase family in tumor vasculogenic mimicry.*J Clin Exp Pathol* 27(11):1181-1184
162. Zhao JH,Zhuang WZ,Long LM,Li F,Liang ZQ (2012) The enhancement of radio-sensitivity via autophagy induced by rapamycin with glioma-initiating cells in vivo.*J. Radiat. Res. Radiat. Process 30*(1):37-41
163. Zhao L,Liu QY,Yin AH,Jiang RQ,Shi J,Wang XH,Sun HC (2010) The inhibitory effect of hydrodynamics-based transfection of IκBα super repressor gene on the orthotopic transplantation tumor model of human hepatocellular carcinoma in in nude mice.*ACTA UNIVERSITATIS MEDICINALIS NANJING（Natural Science）30(6):736*-730,892
164. Zhao Q,Xue CH,Gao Y,Zhang X,Li ZJ,Wang YM,Wang YF (2011) Inhibitory effect and mechanism of echinoside A and ds-echinoside A on growth and metastasis of Lewis lung carcinoma in mice.*Chin J Mar Drugs* 30(5):1-7
165. Zhao X,Tang JY,Xia RM,Yao CG,Guo F,Wang J (2012) The effect of Nanosecond pulsed electric fields on human melanoma A375 cell xenograft in nude mice.*Basic ＆ Clinical Medicine* 32(5):493-499
166. Zhao YX,Wang KW,Yang H,Zheng D,Su ZL,Tong J,Wang SJ,Xu HX (2011) T-bet on lung cancer cell line (Lewis cells) in vivo tumorigenic effects of intervention research. *Ch in J Cell Mol Immunol* 27(8) 862-864
167. Zhao Y,Wang RF,Liu PC,Cui YG (2010) Inhibitory Effect of 125I Seed Interstitial Brachytherapy on Human Hepatocarcinoma Cell HepG2 Transplanted Tumor in Nude Mice.*Journal of Oncology*16(6):446-448
168. Zhao ZD,Shi ZJ,Yang LB,Guan M,Li P,Xiao J,Wang J (2011) Study on the anti-angiogenic activity of tumstatin related peptide T3 mediated by short peptide to osteosarcoma vascular.*Chinese Journal of Orthopaedics* 31(6):699-705
169. Zheng HG,Piao BK,Hua BJ,Zhou YM,Xiong L,Lin HS,Hou W,Pei YX,Qi X (2010) Effect of Feiliuping Ointment and Its Disassembled Prescriptions on Dendritic Cell of Mice with Transplanted Lewis Lung Cancer.*Chinese Journal of Integrated Traditional and Western Medicine* 30(12):12*88*-1291
170. .Zheng LX,Lin DM,Liu HN,Yu SP (2010) The inhibition of mechanism of mouse spontaneous breast cancer growth by nourishing yin and classical prescription by TGF- beta pathway. Chinese Traditional Patent Medicine 33(10):1793-1795
171. Zheng Q,Fang D,Liu M,Jiang H,Cai J,Xing GG (2011) Studies on the excitability of the primary afferent sensory neurons in a rat bone cancer painmodel*.Chinese Journal of Pain Medicine* 17(3):166-170
172. Zheng SP,Wong ZH,Zheng SJ,Guo JL,Wang L,Xie MX (2010) Evaluation of Ant-i tumor Angiogenic Effect of Recombinant Protein Vaccine Combined with Low-dose Gemcitabine in Mice by eFlow Imaging. *Act a Med Uni v Sci Technol Huazhong*39(5):684-687
173. Zheng XY,Yang JP,Hu JH,Wang LN,Jia XM,Yin W,Xu QN,Wang XY,Zuo JL (2012) Relief of mechanical allodynia in rat model of bone cancer by intrathecal MCP-1 neutralizing antibody. *Chinese Pharmacological Bulletin* 28(6):769-772
174. Zheng C,Wang JY,Wang Q,Xu L,Xu ZH (2012) Effects of Chinese herbal medicine Feiyanning decoction on expressions of nucleosome conformation-regulating factors H3-K56,Rtt109, Asf1 and E2F1 in Lewis-bearing mice. *Journal of Chinese Integrative Medicine* 10(4):448-453
175. Zhong H,Luo CL,Zhang AJ,Zhao X,Zhou RY,Liu H (2011) Effect of Bushen Jianpi Decoction and Its Disassemble Recipes on Tumor Growth in M ice with Transplanted Primary Hepatic Carcinoma.*Chinese Journal of Integrated Traditional and Western Medicine* 31(2):213-217
176. Zhou BG,Qiu XC,Xu YM,Fan YX (2010) Pro-apoptotic effect on osteosarcoma SOSP-9607 cells by human recombinant caspase-6 fusion protein.*Chinese Journal of Oncolo*gy 32(7):497-500
177. Zhou HK,Yang DH,Tang SH,Huang W,Jiang XW (2010) Growth Inhibition of Hepatocarcinoma in Nude Mice by Adenovirus-Mediated Transfer of Thymidine Kinase Gene Driven by IGF-II P3 Promoter.*Chinese Journal of Clinical Oncology* 38(12):661-664
178. Zhou J,Xu Y,Zhang MH,Hu,YD (2010) Intratumoral injection of recombinant lentivirus-mediated Bmil-shRNA inhibits growth of A549 xenografts in mice.*ACTA ACADEM IAE MEDIC INAE M ILITARIS TERTIAE* 32(20):2177-2180
179. Zhou J,Yang JP,Ren CG,Li W,Wang LN,Xu QN,Wang XY,Zuo JL (2010) Expression Changes of pCaMK Ⅱ in Spinal Dorsal Horn in a Rat Model of Bone Cancer Pain. *Journal of SOOCHOW university medical science edition* 30(2):233-235
180. Zhou SJ,Liu CA,Gong JP,Liu ZJ,Tang Y,Li SW,Xu Y,Ai ZG (2010) Effect of ultrasound microbubble carring herpes simplex virus thymidine kinase on hepatocellular carcinoma in mice.*Chinese Journal of Hepatology* 18(4):276-279
181. Zhou W,Li YJ,Wei PK (2011) Effects of Xiaotan Sanjie Recipe on Vasculogenic Mimicry of Human Gastric Cancer Xenografts in Nude Mice.*Chinese Journal of Integrated Traditional and Western Medicine* 31(4):532-536
182. Zhou YY,Feng Y,Cao JP,Zhang XG,Zhu W,Ni QY,Geng C,Chen GL,Luo JD (2011) Effects of artesunate combining with radiation on apoptosis in nude mice transplanted with HeLa cells of cervical cancer. *Journal of SOOCHOW university medical science edition* 31(1):13-15
183. Zhou YY,Feng Y,Zhu W,Ni QY,Luo JD,Fan SJ,Cao J (2011) Radiosensitizing effect of artesunate on nude mice transplanted with HeLa cells of cervical cancer.*Chinese Journal of Radiological Medicine and Protection* 31(5):523-525
184. Zhu CF,Sun P,Zhong Q,Gu Y (2011) Studies on effects of human recombinant growth hormone on xenograft hepatocellular carcinoma in nude mice.*J Surg Concepts Pratt* 16(2):176-180
185. Zong WK,Sun BC,SunT,Dong XY,Liu YR,Gu Q,Zhao XL (2011) Study on the Correlation between Caspase8, 9 and EMT Regulating Proteins Twist1 in the Early Stage of Tumor Microenvironment.*Tianjin Med J* 39(4):349-392
186. Zou LP,Luan GX,Feng TT,Xu YJ,Wu CF (2011) Anticancer activities of a new targeting drug VEGF-hFe.*Journal of Siehuan University(Natural Science Edition)* 48(4):929-934
187. Zou XL,Li DH,Wang YS,Zheng JP (2011) The effects of li chong decoction on uterine leiomyoma cell ultrastructure in rats.*Guiding Journal of Traditional Chinese Medicine and Pharmacy* 17(11):3-6
188. Zhang SX,Wu HG,Yao ZA,Wang FF,Du YG (2012) Anti-tumor effect and immunoregulatory activity of two marine oligosaccharides on S180 sarcoma in mice.Curr*ent Immunology* 32(1):5-8
189. Xie Y,Chang H,Shu FR,Zhao CJ,Mi MT (2012) Effects of 3，6-dihydroxyflavone on growth and microRNAs expression profile in transplanted tumor of breast cancer MDA-MB-453 cells.*J Third Mil Med Univ* 34(2):158-160
190. Sun ST,Yang HY,Luo J,Zhu GG,Lai YM,Yang JH (2012) The tumor immunity effect of 4 -1BBL on human PBL -SCID OSCC chimeric model.*China Journal of Oral and Maxillofacial Surgery* 10(5):354-358
191. Tang XL，Fu JH，PARK H，Yang XY (2012) Efficacy and Toxicity of 5-Fluorouracil in Colon Cancer Treatment of a Mouse Syngeneic Model.*Journal of South China Agricuhural University* 33(4):535-538
192. Sun LJ, Shao GQ, Wang ZZ, Zhu XS, Xu LB, Zhao J (2012)Therapeutic effect and distribution of“P-chromic-poly(L-lactide)brachytherapy in nude mice bearing human prostate cancer .*Chinese Journal of Nuclear Medicine* 32(6): 457-462
193. Mai L, Yang L ,Kuang JY, Zhang SQ, Kang YH, Xu QH, Xie JF (2012)Small interfering RNA targeting to hepatitis B virus X gene and 5-aza-2’-deOxycytidineon inhibite growth of the subcutaneous jmplanted tumor of hepatocellular carcinoma in nude mice.*Chinese Journal of Experimental and Clinical Virology* 26(5):362-365
194. Ma JJ, Shen XD, Wang D, Zhang JJ, Chen LZ(2012)Effects of enhancer and Antagonist of Neuropeptide Y On pituitary adenoma of rats . *Chinese journal of neurosurgery* 28(7):733-738
195. Chen XT,Wang YL, Yang Z, Wang JQ(2012)Effect of silencing PARG gene on the growth and metastasis of murine CT26 colon cancer cell xenografted tumors. *Fudan journal (version of medicine)* 39(4):348-359
196. Yang Y, Wang YL, Wang QJ, Chen YT(2012) Effect of Poly( ADP-ribose) glycohydrolase( PARG) genesilencingonliver metastasisof colorectal carcinoma CT26 cell lineinmice.*Basic＆Clinical Medicin* 32（10）：1133-1136
197. Wang L, Wang RW, Jiang YG, Zhao YP,Gong TQ, Guo W (2012)Effects of Src tyrosine kinase inhibitor dasatinib on the proliferation and apopmsb of human esophageal squamous cell carcinoma cell line KYSE. *Oncology* 180 32(7):483-488
198. Wang SJ, Wei AJ , Zhang YQ, Lu HS, Lin YJ, Han HT (2012) AitongxiaoRecipeRegulatedSurvivinandBcl-2 in Rats' transplanted Hepatoma Carcinoma Cell. *Chinese Journal of Integrated Traditional and Western Medicine* 32(12):1652-1657
199. Wang SJ, Wei AJ , Zhang YQ, Lu HS, Lin YJ, Han HT (2012) Effect of Blood Circulation Herb in Aitongxiao Major Prescription on Fas，FasL and Caspase一3 Protein Expression in Transplanted HepatoceUular Carcinoma Cells of Rats.*Lishizhen Medicine and Materia Medica Research* 23(10):2401-2403
200. Liu MH, Li M, Sun Q, Xiao SH (2012) Effectsof Fructopyrano-( 1 >4) glucopyranoseExtractedfromRadixIsatidis on Tumor Growth and Immune Functionin Tumor-BearingMice. *Chinese Pharmaceutical Journal* 47(19):1542-1546
201. Ye H, Guo M, Hao Q, Zhu YZ, Zhen XB (2012) Effect of Scutellaria barbata Polysaccharides on caspase-3,8,9 activity in C26 tumor bearing mice. *Chinese Journal of Gerontology* 32(23):5152-5153
202. Jia YY, Yin YL, Tan WJ, Fu H, Chen X, Pan ZM, Jiao XY (2012) Protective immune responses induced by a recombinant Listeria monocytogenes delivering HPV16 E7, *Acta Microbiologica Sinica* 52（12）：1508-1514
203. Ou BN, Lin XZ, Liang G, Sun YW (2012) The－Epigallocatechin Gallateand Adriamycin Apoptosisof BEL－7404/ ADR in vivo. *Lishizhen Medicine and Materia Medica Research* 23（8）:1854-1855
204. Ou BN, Zhou HD, Liang G,Sun YW (2012) TheReversal Activityof AcEGCG in vivo. *Lishizhen Medicine and Materia Medica Resea*rch 23（9）:2116-2118
205. Pan ZQ, Fang ZQ, Lu WL, Liu XM, Liang C, Wu ZH, Zhang YY (2012) Analysis of the Dynamic Changes of Blood Hormone Levelsin H22 Liver Cancer Mice of Poisonous Pathogenic Factors Syndromesto Different Degrees. *Chinese Journal of Integrated Traditional and Western Medicine* 32（10）:1361-1365
206. Zhang Y, Lu H, Sun XW,Fan ZZ, Sun J, Xu JH（2012) Effects of Chang wei qing inrever sing oxaliplatin resistant HCT116/L-OHP and Influence on Pt-DNA adduct.*Chinese Traditional Patent Medicine* 34（10）:1843-1848
207. Gao RQ, Zhou XY, Yang YX, Wang ZG (2012) Inhibition of suhretinal CO-transfection of Rb94 and wild—type p53 gene on retinoblastoma by ultrasound microbubble in nude mouse.*Chinese Journal of Experimental Ophthalmology* 30 (10) :908-913
208. Wang WJ, Li Hai, Du Y, Zhang D, Yang YX (2012) Inhibitory effects of celecoxib on growth of colorectal cancer in experimental rats.*Chinese Journal of Experimental Surgery* 29（7）:1328-1331
209. Wang Y, Li LJ, Xu K, Lv P, Zhen WL (2012) In vivo study on antisense-micro ribonucleic acid-21 oligonucleotide inhibiting tongue squamous cell carcino—ma growth. *West China Journal of Stomatology* 30（6）:562-567
210. Shen KP, Wang HB, Hu B, Liu W (2012) Effectsof Wei －Chang－Anonthe Expression of Epithelial Mesenchymal Transition Related Genes in SGC－7901 Gastric Carcinoma.*Lishizhen Medicine and Materia Medica Research* 2（4）:846-847
211. Pan Y, Hua YQ, Liu LM, Shen JG (2012) Isolation and Identification of Pancreatic Cancer Stem Cells from BxPC 3 Cell Line Cultured in Serum-Free Medium.*Chinese Journal of Oncology* 21（6）:441-445
212. Wang LF, Meng FR, Zhou Y, Cao Q, Shan BE (2012) Effect of triterpenes compound of cortex periplocae on PCNA expression in rat esophageal carcinoma.*Chinese Journal of Cancer Biotherapy* 10（5）:508-512
213. Wang LF, Lu A, Meng FR, Cao Q, JI X, Shan BE (2012) Inhibitory Effects of Triterpenes Compound of Cortex Periplocae on N-nitrosomethylbenzylamine-induced Rat Esophageal Tumorigenesis.*Cancer Prevention Research* 39（1):23-27
214. Xu F, Zhang L, Li J, Zhao L, Qian NS (2012) The changes of GluR1 expression in the central amygdala of mouseis correlated to the bone cancer pain.*Chinese Journal of Neuroanatomy* 28(5) :501～506
215. Zhou HY, Meng ZY, Dou GF, He TQ, Lou YQ, Zhang GL (2012) Study on tissue distribution of1，2-［bis(1，2-benzisoselenazolone-3(2 )-ketone) -ethane in nude mice and SDVrats*.Chinese Journal of New Drugs* 21（21）:2359-2343
216. He XH, Wang LJ, Zhou M, Han RC, Zhen Q, Yang YP, Sha YL (2012) Feasibility of in Vivo Imaging using Bifido bacterium bifidum as a Carrier for Delivering Quantum Dots intoTumors.*Chinese Journal of Biochemistry and Molecular Biology* 28（9）:864-869
217. Li XT, Zhao L, Tan JJ, Cheng L.Comparison on antitumor effects of different vinorelbine preparations. *Chinese Traditional and Herbal Drugs* 43（9）:1794-1798
218. Yu B, Wang YR, Yin XR, Tang W.Effects of Bifidobacterium Infantismediated TK/GCV Suicide Gene Therapy System on Bladder Cancer Cell Apoptosis and Fas/FasL Ligand Expression in Rats.*Journal of China Medical University* 41（10）:877-881
219. Xin B, Wang XY, Li Y, Qin JH, Ma XJ, Yin JP, Wang RA (2012) Expression and potential role of metastasis-associated protein 1 in the induced carcinogenesis of mouse live.*Chin J Cell Mol Immunol* 28（8）:801-803
220. Sun YL, Xu C, Su CQ, Ma JX, Wu HY (2012) The immune function effect of adenovirus-mediated HSP70 gene expression on tumor-bearing mice of pancreatic cancer.*Chongqing Medical Journal* 41（32）:3353-3359
221. Chen XH, Jiang FF, Liu B,Wu XL, Yu DS (2012) Radiation-inducible promoters mediated PUMA gene in the treatment of enograft of human tongue squamous in naked mice.*Chinese journal of dental research* 6（5）:421-425
222. Li SF, Liu DX, Zheng YH, Yue Y, Huang YB, Jing XB (2012) Inhibitory effe cts of paclitaxel-loadpla nanofibers against mice cervical cancers.*Acta Polymerica* Sinica 9（9）:1029-1034
223. Zhao X, Xue CH, Yang YH, Xue Y, Wang YM, Li ZJ (2012) Inhibitory Effect of Echinoside A and Ds-Echinoside A on Spontaneous Lung Metastases of B16 Melanoma Cells in C57BL/6 Mice.*Journal of Food Science* 33（9）:230-234
224. Zhang X, Wang JF, Xu L, Wang YF, Liu D, Xue CH, Li ZJ (2012) Comparative Anti-tumor Effects of Fucoidan from Two Sea Cucumber Species on Spontaneous Metastasis of Lewis Lung Carcinoma in Mouse.*Journal of Food Science* 33（7）:251-255
225. Dong H, Zhen GQ, Teng AG, Zhang QY, Chen J, Fang JJ, Liu AJ (2012) Mechanism of splenic Excess Augmentation in tumor-bearing Mice.*Cancer Prevention Research* 39（8）:940-943
226. Sun X, Zhao XH, Bao HY (2012) Antitumor Active Constituent in Fruiting Body of Fomitopsis pinicola.*Lishizhen Medicine and Materia Medica Research* 23（7）:1634-1637
227. Peng Y, Song XL, Shen MH （2012）Inhibitory Effect ofHericium erinaceusPolysaccharide on Hepatoma-22 (H22) Tumor Bearing Mice.*Journal of Food Science* 33（9）:244-246
228. Yang BL, Chen HJ, Chen YG, Gu YF, Zhang SP, Lin Q, Zhu P (2012) Effects of Baicalin on an orthotopic transplantation mouse model of mismatch repair gene deficient colorectal cancer. *Chinese Journal of Surgery*  50（9）:843-847
229. Ma K, Ma ZG, Zhang LN,Zhou GP,Bian J, Zhou H (2012) Effects of Hui medicine Aikang Fang for lewis lung carcinoma inhibition and mutant p53 expression.*Journal of Chinese medicine* 27（8）:2158-2160
230. Liu S, He LL, Zhang LN, Sun GZ (2012) Effects of chinese drugs for activating blood and chinese drugs for nourishing oi and activating blood on the metastasis of lewislung carcinoma in different Stages.*Chinese Journal of Integrative Medicine* 32（12）:1638-1641
231. Lian M, Jiang LY, Wang h, Fan EZ, Wang Q, Fang JG (2012) Inhibition of human Blaryngeal carcinoma growth by gene therapy and epigenetic therapy. *Chinese Journal of Otorhinolaryngology Head and Neck Surgery* 47(9）:753-759
232. Jiang SL, Chen BA, Wen J (2012) Radiationtherapyeffect of extremely low frequency fields combined with X-ray on hepatoma-implanted mice. *Journal of southeast university* (medical edition) 31（6）:670-673
233. Li CJ, Ke,CB, Shi D, He WS, Bu HL, Gao F, Tian YK (2012) Role of protocadherin 20 in spinal cord in devlopment of bone cancer pain in rats.*Chinese Journal of Anesthesiology* 31（2):1445-1448
234. Zhao QQ, Du QH, Wang SL, Wang GM, Zhang MY (2012) Hyperalgesia in rats with tibial cancer pain: formation and Possible mechanisms. *Shandong University* 50（7）:38-40
235. Wang R, Xiao X, Lin Y, Zhang L, Wang XJ (2012) Effects of ardipusilloside analogs on human glioma cell strain U373 xenograft Balb/c nude mouse tumor.*Chinese Traditional Patent Medicine* 34（1）:2051-2054
236. Geng X, Huang J, Lao X, Wu ZR (2012) Study on the in vivo toxicity and antitumor activity of poly (- glutamic acida ) asp-cisplatin complex. *Chinese Journal of Hospital Pharmacy* 32（13）:1005-1008
237. Lu D, Miao LF, Liu SS, Xiong QY, Guo F (2012) Inhibitory effect of lanthanum chloride on mice transplanted tumor of uterine cervical cancer .*Chinese Journal of Obstetrics and Gynecology* 47(7) :540-542
238. Chen Y, Yang GM, Zhou YC, Liu X, Jin CG, Chen XQ, Li J, Wang XC (2012) Gene silencing by lentivirus-mediated delivery of integrin β4 shRNA inhibits the growth of subcutaneous xenografts of human non-small cell lung carcinoma H460SM cells in nude mice.*Tumor* 32（8）; 578-584
239. Yao CG, Guo F, Wang J, Sun XC, Zhao X, Tang JY (2012) Lo n g — t e r m Eff ects of Nanosecond Pulsed Electric Field Oll Nude Mouse Model with Human Melanoma. *High Voltage Engineering* 38（12）:3357-3362
240. Huang XP, Zhang J, Zhang MP, Liu B, Yang JJ,Wen Q, Luo FH, ZHuang GH (2012) Preparation and identification of aDR5ScFv loaded GCS and assaying its antitumor effect in vivo. *Journal of Immunology* 28（9）:750-754
241. Wu ZY, Li FF, Zhang K, Zhu CL, Wang Y, Du J (2012) Effect of artificial Bovis Calculus on pulmonary metastasis from breast cancer of mice .*Chinese Traditonal drug* 43（10）:2013-2016
242. Qiu F, Yuan WX, Mi WD, Wei XL, Liu YH (2012) Changes in expression of acid-sensing ion channel 3 in dorsal root ganglion in a rat model of bone cancer pain. *Chinese Journal of Anesthesiology* 32（10）:1214-1217
